# HapX-mediated H2B deub1 and SreA-mediated H2A.Z deposition coordinate in fungal iron resistance

**DOI:** 10.1093/nar/gkad708

**Published:** 2023-08-31

**Authors:** Kewei Sun, Yiqing Li, Yunpeng Gai, Jingrui Wang, Yunqing Jian, Xin Liu, Liang Wu, Won-Bo Shim, Yin-Won Lee, Zhonghua Ma, Hubertus Haas, Yanni Yin

**Affiliations:** State Key Laboratory of Rice Biology, the Key Laboratory of Molecular Biology of Crop Pathogens and Insects, Institute of Biotechnology, Zhejiang University, Hangzhou, China; State Key Laboratory of Rice Biology, the Key Laboratory of Molecular Biology of Crop Pathogens and Insects, Institute of Biotechnology, Zhejiang University, Hangzhou, China; School of Grassland Science, Beijing Forestry University, Beijing, China; State Key Laboratory of Rice Biology, the Key Laboratory of Molecular Biology of Crop Pathogens and Insects, Institute of Biotechnology, Zhejiang University, Hangzhou, China; State Key Laboratory of Rice Biology, the Key Laboratory of Molecular Biology of Crop Pathogens and Insects, Institute of Biotechnology, Zhejiang University, Hangzhou, China; Institute of Food Safety and Nutrition, Jiangsu Academy of Agricultural Sciences, Nanjing, China; Institute of Crop Science, Zhejiang University, Hangzhou, China; Department of Plant Pathology and Microbiology, Texas A&M University, College Station, USA; Department of Agricultural Biotechnology, Seoul National University, Seoul, Korea; State Key Laboratory of Rice Biology, the Key Laboratory of Molecular Biology of Crop Pathogens and Insects, Institute of Biotechnology, Zhejiang University, Hangzhou, China; Instiute of Molecular Biology, Biocenter, Medical University Innsbruck, Innsbruck A-6020, Austria; State Key Laboratory of Rice Biology, the Key Laboratory of Molecular Biology of Crop Pathogens and Insects, Institute of Biotechnology, Zhejiang University, Hangzhou, China

## Abstract

Plant pathogens are challenged by host-derived iron starvation or excess during infection, but the mechanism through which pathogens counteract iron stress is unclear. Here, we found that *Fusarium graminearum* encounters iron excess during the colonization of wheat heads. Deletion of heme activator protein X (FgHapX), siderophore transcription factor A (FgSreA) or both attenuated virulence. Further, we found that FgHapX activates iron storage under iron excess by promoting histone H2B deubiquitination (H2B deub1) at the promoter of the responsible gene. Meanwhile, FgSreA is shown to inhibit genes mediating iron acquisition during iron excess by facilitating the deposition of histone variant H2A.Z and histone 3 lysine 27 trimethylation (H3K27 me3) at the first nucleosome after the transcription start site. In addition, the monothiol glutaredoxin FgGrx4 is responsible for iron sensing and control of the transcriptional activity of FgHapX and FgSreA via modulation of their enrichment at target genes and recruitment of epigenetic regulators, respectively. Taken together, our findings elucidated the molecular mechanisms for adaptation to iron excess mediated by FgHapX and FgSreA during infection in *F. graminearum* and provide novel insights into regulation of iron homeostasis at the chromatin level in eukaryotes.

## Graphical Abstract

**Graphical Abstract:**
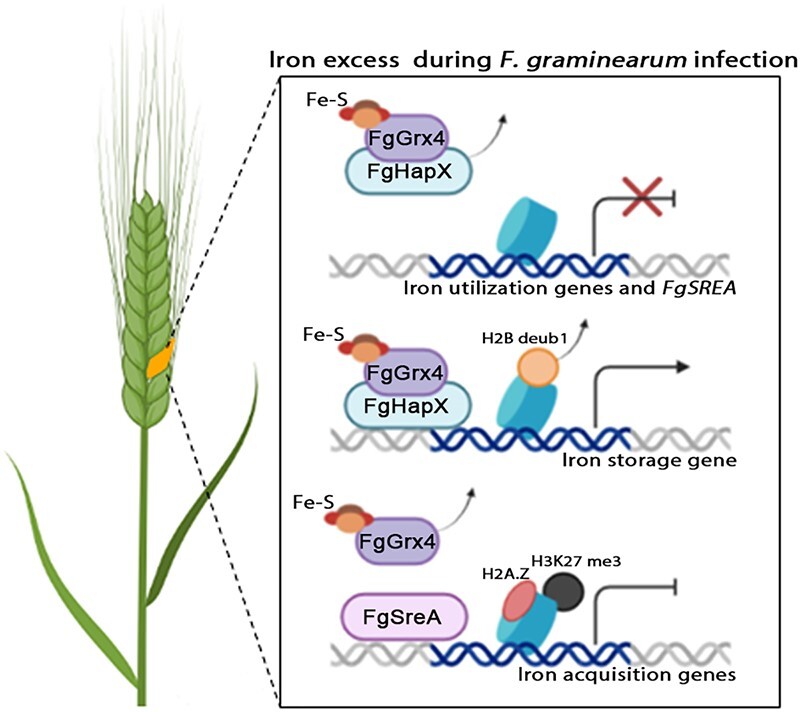

## INTRODUCTION

Iron is essential for almost all organisms because of its roles in oxygen delivery, electron transport, and enzymatic activity. However, iron overload can lead to the production of toxic reactive species through the Fenton reaction, which can damage various cellular components (1–3). Therefore, adaptation to iron starvation and excess is important for cell survival in dynamically changing growth niches. In the interactions between hosts and pathogens, hosts usually use iron-withholding strategies to restrict the growth of invading pathogens (4–6). Compared to plants grown under iron-sufficient conditions, iron-deprived *Arabidopsis* displayed increased resistance to attacks by the bacterium *Dickeya dadantii* and the fungus *Botrytis cinerea* (7). Iron acquisition has been found to be essential for virulence of the mammalian pathogens *Aspergillus fumigatus* (8), *Cryptococcus neoformans* (9), and *Candida albicans* (10), as well as the plant pathogens *Xanthomonas* (11), *Ustilago maydis* (12) and *Fusarium oxysporum* (13). In contrast to iron-withholding strategies, some plants resist pathogen infection by locally elevating iron levels to trigger toxic oxidative burst. For example, wheat redistributes ferric iron to the apoplast of epidermal leaf cells and then induces hydrogen peroxide (H_2_O_2_) production to defend against *Blumeria graminis* invasion (14). Similarly, the pathogens *Verticillium dahlia* and *Colletotrichum graminicola* are challenged by iron excess during infection (15). However, the molecular mechanisms by which pathogens overcome iron starvation or iron excess during successful infection remain to be fully characterized.

All organisms have developed sophisticated strategies to maintain intracellular iron homeostasis via fine-tuning of iron acquisition, utilization, and storage. Fungi acquire iron from their habitats by employing a variety of complex strategies such as siderophore-mediated iron uptake (SIM), reductive iron assimilation (RIA), and utilization of heme as iron source (16,17). Therefore, the regulatory mechanisms of iron homeostasis in fungi are different from those in mammals. In mammals, intracellular iron homeostasis is mainly controlled through the coordinated post-transcriptional regulation of diverse iron metabolism genes (18). In filamentous fungi, iron homeostasis is primarily maintained via transcriptional regulation mediated by two transcription factors, including the bZIP transcription factor HapX (heme activator protein X) and the GATA zinc finger transcription factor SreA (siderophore transcription factor A) (19–22). The roles of HapX and SreA in iron homeostasis have been well characterized in *A. fumigatus*. During iron starvation, HapX represses iron utilization pathways and activates high-affinity iron acquisition including SIM and RIA (8,23). During periods of iron sufficiency, SreA represses high-affinity iron acquisition including RIA and SIM (23,24). HapX is also critical for iron detoxification by triggering the expression of the vacuolar iron importer CccA (cross-complements Ca^2+^ phenotype of csgA) under conditions of iron excess (23,25). HapX and SreA are interconnected by a negative transcriptional feedback loop (26); i.e. under iron starvation HapX represses SreA and under iron sufficiency SreA represses HapX. Recently, the complex promoter target recognition of iron repressed genes by HapX was elucidated at the molecular level (27). However, the detailed mechanisms of HapX and SreA in regulating the transcription of iron homeostasis-maintaining genes remain unclear.

Apart from transcription factors, chromatin structure modulated by histone modifications or remodeling complexes is a major transcriptional regulator in eukaryotes (28–30). Although the intricate transcriptional network of iron homeostasis has been extensively studied in plants and fungi (31–34), the impact of chromatin structure on expression of genes involved in maintenance of iron homeostasis has not been well characterized. Recently, histone 3 lysine 27 trimethylation (H3K27 me3) was found to repress the transcription of iron acquisition genes to prevent iron overload in *A. thaliana* (35). In addition, the Spt-Ada-Gcn5-acetyltransferase (SAGA) chromatin-modifying complex is involved in regulating iron homeostasis in *C. albicans* and *F. graminearum* (36,37). To the best of our knowledge, there have been no other reports on regulation of fungal iron homeostasis at the chromatin level.

Fusarium head blight (FHB) is an economically devastating disease of small grain cereal crops mainly caused by *F. graminearum* (38). In addition to causing yield loss, *F. graminearum* also contaminates grains with mycotoxins, such as deoxynivalenol (DON) and zearalenone, which pose a great threat to human and animal health (39–41). More recently, pH-dependent transcription factor FgPacC prevents histone acetylation of iron acquisition genes, and then reduces their transcription in response to iron excess under alkaline host microenvironment during *F. graminearum* infection (37).

Here, we found that *F. graminearum* encounters iron excess before host alkalinization during the infection of wheat spikelets and coleoptiles, therefore it was intriguing to explore how this fungus overcome iron excess under non-alkaline host microenvironment. Our study revealed that (i) the iron-responsive transcription factors FgHapX and FgSreA play important roles in iron resistance before host alkalinization during infection, (ii) FgHapX enhances histone H2B deubiquitination (H2B deub1) in the promoter of *FgCCCA* to activate iron storage under iron excess, (iii) FgSreA promotes the deposition of histone variant H2A.Z and the H3K27 me3 level at iron uptake genes to decrease transcription under iron excess, and (iv) the monothiol glutaredoxin FgGrx4 senses and transmits the cellular iron status to FgHapX and FgSreA. Overall, our results elucidated the molecular mechanisms for overcoming iron excess mediated by FgHapX and FgSreA during infection in *F. graminearum*, providing novel insights into regulation of iron homeostasis at the chromatin level in eukaryotic organisms and broaden our knowledge on iron stress and virulence during phytopathogen-host interactions.

## MATERIALS AND METHODS

### Strains and sensitivity determination

The wild-type strain of *F. graminearum* PH-1 was used as the parental strain for the transformation experiments. To determine sensitivity to iron and oxidative stresses, 5-mm mycelial plugs of each strain taken from a 3-day-old colony edge were inoculated on complete medium (CM; 50 ml nitrate salts [120 g NaNO_3_, 10.4 g KCl, 10.4 g MgSO_4_•7H_2_O, 30.4 g KH_2_PO_4_, per 1 l], 1 g casamino acid, 10 g D-glucose, 2 g peptone, 1 g yeast extract, 1 ml trace element [2.2 g ZnSO_4_•7H_2_O, 1.1 g H_3_BO_3_, 0.5 g MnCl_2_•4H_2_O, 0.5 g FeSO_4_•7H_2_O, 0.17 g CoCl_2_•6H_2_O, 0.16 g CuSO_4_•5H_2_O, 0.15 g Na_2_MoO_4_•5H_2_O, 5 g Na_4_EDTA, per 100 ml], 1 ml vitamin solution [0.01 g biotin, 0.01 g pyridoxin, 0.01 g thiamine, 0.01 g riboflavin, 0.01 g p-aminobenzonic acid, 0.01 g nicotinic acid, per 100 ml] and 10 g agar, 1 l ddH_2_O, pH 7.0) or minimal medium (MM; 0.5 g KCl, 2 g NaNO_3_, 1 g KH_2_PO_4_, 0.5 g MgSO_4_.7H_2_O, 0.01 g FeSO_4_.7H_2_O, 30 g sucrose, 200 μl trace element solution [5 g citric acid, 5 g ZnSO_4_.7H_2_O, 1 g Fe(NH_4_)_2_(SO_4_)_2_•6H_2_O, 0.25 g CuSO_4_•5H_2_O, per 100 ml] and 10 g agar, 1 l ddH_2_O, pH 7.0) supplemented with FeSO_4_ and H_2_O_2_, respectively, and then incubated at 25°C for 3 days in the dark. The concentrations of each compound were indicated in the figure legends. The mycelial growth inhibition rate % = [(*C* − *N*)/*C*] × 100, where *C* is the colony diameter of the control without treatment and *N* is that with treatment. The colony diameter was subtracted by 0.5 cm (initial size of hyphae). Three technical replicates were used for each strain and each experiment was repeated three times independently.

### Determination of iron content

To microscopically examine the iron content, each strain was stained with 5 μM FeRhoNox-1 (Goryo Chemical, Inc., Sapporo, Japan) for 1 h after culture in CM for 36 h (42,43). Then, fluorescence signals were examined using a Zeiss LSM780 confocal microscope (Gottingen, Niedersachsen, Germany). To quantify the iron content, approximately 50 mg of fresh mycelia cultured in CM were lysed using 2% cellulase (Ryon Biological Technology CO, Ltd, Shanghai, China), 2% lysozyme (Ryon Biological Technology CO, Ltd, Shanghai, China), and 0.2% driselase from *Basidiomycetes* sp. (Sigma, St. Louis, MO, USA) for 4–6 h. After filtration with funnel and filter paper, the filtrate was centrifuged at 5000 × *g* at 4°C for 10 min and then used for iron content determination. Iron content was determined using a previously reported colorimetric ferrozine-based assay, with ferrozine as a chelator and ferric chloride as a standard (44). Briefly, aliquots (100 μl) of cell lysates were mixed with 100 μl of 10 mM HCl (the solvent of the iron standard FeCl_3_) and 100 μl of the iron-releasing reagent (a freshly mixed solution of equal volumes of 1.4 M HCl and 4.5% KMnO_4_ in ddH_2_O). The mixtures were incubated in a fume hood at 60°C for 2 h. After the mixtures had cooled to room temperature, 30 μl of the iron-detection reagent (6.5 mM ferrozine, 6.5 mM neocuproine, and 2.5 M ammonium acetate and 1 M ascorbic acid) was added to each tube. After 30 min, 200 μl of the solution from each tube was transferred into a well of a 96-well plate, and the absorbance was measured at 550 nm using a microplate reader. The linear range of the ferrozine assay ranged from 0.2 to 30 nmol. Three technical replicates were used for each strain and each experiment was repeated three times independently.

### Determination of alkalinization at the infection sites

Wheat coleoptiles and seedling leaves were placed on water agar medium plates (adjusted to pH 5.0) containing bromothymol blue or phenol red, and then inoculated with the conidial suspension of PH-1 or water as a control. Plate were cultured at 25°C for 7 days, and were imaged each day. For bromothymol blue staining, bright yellow indicates pH < 5.2, deep purple indicates pH > 6.8. For phenol red staining, bright yellow indicates pH < 6.8, red indicates pH > 8.0. Three technical replicates were used for each strain and each experiment was repeated three times independently.

To determine pH in apoplastic fluids, wheat coleoptiles and seedling leaves inoculated with *F. graminearum* at different time intervals were washed and vacuum infiltrated with cold water twice for 2 min. Then apoplastic fluids were collected by centrifugation at 3000 × *g* for 5 min, and the pH of apoplastic fluids was measured with a pH electrode (model PHM93, Radiometer Analytical).

### Determination of virulence, DON production, and conidiation

To evaluate the virulence of wheat spikelets, a 10 μl aliquot of conidial suspension (10^5^ conidia ml^−1^) was injected into a floret in the central section spikelet of single flowering wheat heads of susceptible wheat (*Triticum aestivum*) cultivar Jimai22. Each strain had 20 replicates. At the 15 days post inoculation (dpi), the number of infected spikelets in each inoculated wheat head was recorded, and this number was used to determine the disease index of PH-1 and the mutants.

For coleoptile infection assay, seeds of Jimai22 were randomly planted and cultivated under the same conditions. A 10 μl aliquot of conidial suspension (10^5^ conidia ml^−1^) was inoculated on coleoptiles as described in the previous study (45). Lesion sizes on the coleoptiles were measured at 4 dpi. At least 10 infected wheat heads or coleoptiles were examined for each strain, and each virulence experiment was repeated three times.

To determine DON production, vegetative hyphae of each strain were collected from a 24 h yeast extract peptone dextrose (YEPD; 10 g peptone, 3 g yeast extract and 20 g glucose, 1 l ddH_2_O, pH 6.7) culture and then inoculated into trichothecene biosynthesis induction medium (TBI; 30 g sucrose, 1 g KH_2_PSO_4_, 0.5 g MgSO_4_•7H_2_O, 0.5 g KCl, 0.01 g FeSO_4_•7H_2_O, 0.8 g putrescine salts and 200 ul trace element [5 g citric acid, 5 g ZnSO_4_•7H_2_O, 0.25 g CuSO_4_•5H_2_O, 0.05 g MnSO_4_•5H_2_O, 0.05 g H_3_BO_3_, per 100 ml], 1 l ddH_2_O, pH 4.5) liquid medium at 28°C for 7 days in a shaker (150 rpm) in the dark (46). Then, the DON production for each sample was extracted and quantified using a DON Quantification Kit Wis008 (Wise Science, Zhenjiang, China). The dry weight of the mycelia was used as an internal reference. Three technical replicates were used for each strain and each experiment was repeated three times independently.

For the conidiation assay, fresh mycelia (50 mg) of each strain were inoculated in a 50 ml flask containing 20 ml of carboxymethyl cellulose medium (CMC; 15 g sodium carboxymethyl cellulose, 1 g yeast extract, 1 g NH_4_NO_3_, 1 g KH_2_PO_4_ and 0.5 g MgSO_4_•7H_2_O, per 1 l ddH_2_O, pH 6.5) liquid medium. The flasks were incubated at 25°C for 4 days in a shaker (180 rpm). Subsequently, the number of conidia in each flask was determined using a hemocytometer. Three technical replicates were used for each strain and each experiment was repeated three times independently.

### Quantitative real-time polymerase chain reaction (qRT-PCR) and RNA-seq analysis

Strains were grown in CM for 36 h and then treated with or without 10 mM FeSO_4_. RNA from each strain was extracted using TaKaRa RNAiso Reagent (TaKaRa Biotechnology Co., Dalian, China). Each RNA sample was reverse transcribed using a HiScript II Q RT Kit (Vazyme, R223-01, Nanjing, China). qRT-PCR reactions were performed in 20 μl on CFX Connect™ Optics Module machine (Bio-Rad Laboratories, Shanghai, China) and with the following cycling conditions: 95°C/30 s and then 40 cycles of 95°C/5 s, 60°C/15 s, each reaction was carried out with primers listed in Supplementary Table S1. The comparative cycle quantification (Cq) method was used for data analysis and relative fold difference was expressed as 2^−ΔΔCq^ and the *FgACTIN* gene was used as the internal control for normalization. The transcription of the wild-type without 10 mM FeSO_4_ treatment was set to 1. Two technical replicates were used for each strain and each experiment was repeated three times independently.

For RNA-seq analysis, *F. graminearum* mycelia were collected at 3 and 4 dpi from infected wheat coleoptiles, or were collected from the CM culture with or without iron excess treatment. RNA-seq was conducted using Illumina NovaSeq 6000 (Genergy Bio, Shanghai, China) sequencing system. RNA-Seq by Expectation-Maximization (RSEM) software was used to calculate the values of fragments per kilobase of transcript per million fragments mapped (FPKM) to evaluate gene expression level. DEGs (differentially expressed genes) detection was performed with DEseq2 (Parameters: Fold Change2.00) and adjusted *P* value ≤0.05 as described. Three biological replicates were conducted for each strain.

### Mutant generation

Constructs for gene deletion of *F. graminearum* using a PEG-mediated protoplast transformation method were carried out as described previously (47). Briefly, fresh mycelia were treated with driselase (D9515, Sigma, MO, USA), lysozyme (RM1027, RYON, Shanghai, China) and cellulose (RM1030, RYON, Shanghai, China). Primers used to amplify the flanking sequences for each gene are listed in Supplementary Table S1. For generation of deletion mutants, the open reading frame (ORF) of each gene was replaced with hygromycin resistance cassette (*HPH*), for generation of double deletion mutants ΔFgHapX-FgSreA, the ORF of *FgHAPX* was first replaced with *HPH* cassette, and then *FgSREA* was replaced with a geneticin resistance (*NEO*) cassette (Supplementary Figure S1A). Deletion mutants ΔFgUbp8 (ubiquitin-specific processing protease 8), ΔFgSgf73 (SAGA associated factor 73), ΔFgSwc6 (SWR1 complex subunit 6), ΔFgSwc2 (SWR1 complex subunit 2), ΔFgEed (embryonic ectoderm development protein) and double deletion mutants ΔFgHapX-FgSreA were identified by PCR and Southern blot assays (Supplementary Figure S1B, C). The deletion mutants ΔFgHapX, ΔFgSreA, ΔFgRad6 and ΔFgBre1 were obtained from our previous studies (22,45).

To knock down FgHapB, we replaced the FgHapB native promoter with Pzear (48). The *HPH* and Pzear fragments were amplified and fused by overlap PCR. Subsequently, the ‘*HPH*-Pzear’ fragment was further fused with the 5′ and 3′ flanking regions of the FgHapB native promoter. The resulting fusion fragment was purified and transformed into PH-1 to replace the native promoter of FgHapB (Supplementary Figure S1A). To induce Pzear expression, the inducer β-estradiol (30 μM) was added to the medium during the regeneration and mutant selection processes (48). The knock-down mutants *FgHapB^KD^* were identified by PCR and a Southern blot assay (Supplementary Figure S1B, C). The expression of *FgHapB^KD^* was confirmed using qRT-PCR (Supplementary Figure S1D)

### Flag-, GFP- and RFP-fusion cassettes construction

To construct the FgHapX-GFP cassette, FgHapX containing native promoter region and ORF (without the stop codon) was amplified with the relevant primers in Supplementary Table S1. The resulting PCR products were co-transformed with XhoI-digested pYF11 containing a *NEO* cassette into the yeast strain XK1-25 using the Alkali-Cation Yeast Transformation Kit (MP Biomedicals, Solon, USA) to generate the recombined FgHapX-GFP fusion vector. Subsequently, the FgHapX-GFP fusion vector was recovered from the yeast transformant using the Yeast Plasmid Kit (Solarbio, Beijing, China) and then transferred into *Escherichia coli* strain DH5α for amplification. Using the similar strategy, FgHapX-Flag, FgSreA-GFP and -Flag, FgUbp8-Flag, FgSwc6-GFP and -RFP, FgSwc2-GFP, FgEed-Flag and FgGrx4-GFP fusion cassettes were constructed. The FgHapX-GFP and FgSreA-GFP cassettes were respectively transformed into ΔFgHapX and ΔFgSreA to acquire complemented strains. The FgUbp8-GFP was transformed into ΔFgUbp8 to get strain ΔFgUbp8::FgUbp8-GFP for ChIP-qPCR assays. Cassette pairs FgHapX-GFP + FgUbp8-Flag, FgSwc6-GFP + FgSreA-Flag, FgSwc2-GFP + FgEed-Flag, FgSwc6-GFP + FgEed-Flag, FgGrx4-GFP + FgHapX-Flag, FgGrx4-GFP + FgSreA-Flag were transformed into PH-1 for Co-IP assays. The cassettes FgSreA-GFP and FgSwc6-RFP were transformed into PH-1 for co-localization assay.

To construct the Flag-FgH2A.Z cassette, the RP27 promoter combined with Flag and H2A.Z ORF was amplified with the relevant primers in Supplementary Table S1. The cassette Flag-FgH2A.Z was transformed into PH-1 for checking the specificity of polyclonal anti-H2A.Z antibody (produced by ABclonal^®^ Technology, Wuhan, China).

### Transcriptional activation activity assay

The coding sequence of FgHapX was amplified from the cDNA of PH-1 with primer pairs indicated in Supplementary Table S1, then was fused in frame with the receptor GAL4 DNA-binding domain in a pGBKT7 vector by recombination reactions. The plasmids pGBKT7-FgHapX and pGBKT7 were separately transformed into Y2HGold cells. Transformants were grown at 30°C for 3 days on SD lacking Trp, and then transferred to SD stripped of Trp, His, and containing 1 mM 3-aminotriazole (SD-Trp-His + 3AT) with or without 10 mM FeSO_4_ to assess transcriptional activation activity (49). Three independent experiments were conducted.

### Yeast two-hybrid (Y2H) assays

To construct plasmids for Y2H analyses, the coding sequence of each tested gene was amplified from the cDNA of PH-1 with primer pairs indicated in Supplementary Table S1. The cDNA fragment was inserted into the yeast GAL4-binding domain vector pGBKT7 and the GAL4-activation domain vector pGADT7 (Clontech, Mountain View, CA, USA), respectively. The pairs of Y2H plasmids were co-transformed into *S. cerevisiae* strain Y2HGold following the lithium acetate/single-stranded DNA/polyethylene glycol transformation protocol. In addition, the plasmid pair pGBKT7-53 and pGADT7-T served as positive controls. The plasmid pair pGBKT7-Lam and pGADT7-T was used as negative controls. Transformants were grown at 30°C for 3 days on SD lacking Leu and Trp, and then transferred to SD stripped of Trp, Leu, His, and containing 1 mM 3-aminotriazole (SD-Trp-Leu-His + 3AT) to assess protein-protein interactions. Three independent experiments were performed to confirm Y2H results.

To search for FgHapX-interacting proteins, we performed Y2H screening. FgHapX was cloned into the yeast vector pGBKT7. A *F. graminearum* cDNA library was constructed in the Y2H vector pGADT7 using total RNA extracted from mycelia and conidia under iron excess treatment. Y2HGold, which was co-transformed with the cDNA library, as well as FgHapX-pGBKT7 or FgSwc2- pGBKT7, were directly selected using SD-Trp-Leu-His + 3AT (1 mM). Approximately 500 potential yeast transformants containing cDNA clones interacting with FgHapX was further confirmed in selection medium SD-Trp-Leu-His + 3AT (1 mM).

### Co-immunoprecipitation (co-IP) assays

The GFP- and 3 × Flag-fusion constructs were verified by DNA sequencing and transformed into pairs into PH-1. Transformants expressing a pair of fusion constructs were confirmed by western blot analysis. For Co-IP assays, fresh mycelia (500 mg) of each strain were finely ground and suspended in 1 ml of extraction buffer containing 1% protease inhibitor. After homogenization with a vortex shaker, the lysates were centrifuged at 10 000 × *g* for 20 min at 4°C, and the supernatants were incubated with anti-GFP (ChromoTek, Martinsried, Germany) agarose. Proteins eluted from agarose were analyzed by western blotting with mouse monoclonal anti-Flag (A9044, Sigma, St. Louis, MO, USA) and rabbit polyclonal anti-GFP (ab32146, Abcam, Cambridge, UK) antibodies. Samples were also detected with a mouse monoclonal anti-GAPDH antibody (EM1101, HuaAn Biotech. Ltd, Hangzhou, China) as a reference. After inoculation with the secondary antibody, goat polyclonal anti-rabbit IgG-HRP (HA1001, HuaAn Biotech. Ltd., Hangzhou, China), or goat polyclonal anti-mouse IgG-HRP (HA1006, HuaAn Biotech. Ltd., Hangzhou, China), chemiluminescence was detected. All blots were imaged using the Image Quant LAS4000 mini (GE Healthcare, Chicago, IL, USA). The experiment was conducted three times independently.

### Western blotting assays

*F. graminearum* protein extraction was performed as described in the Co-IP assay. The resulting proteins were separated by 10% sodium dodecyl sulfate-polyacrylamide gel electrophoresis (SDS-PAGE) and transferred to an Immobilon-P transfer membrane (Millipore, Billerica, MA, USA). GFP- and Flag-tagged proteins were detected with monoclonal anti-GFP (ab32146, Abcam, Cambridge, UK) and monoclonal anti-Flag (A9044, Sigma, St. Louis, MO, USA) antibodies, respectively. H2B ub1 and H3K27 me3 levels were detected using monoclonal anti-H2B ub1 (5546, Cell Signaling Technology Inc., Boston, MA, USA) and monoclonal anti-H3K27 me3 (39155, Active Motif, Shanghai, China) antibodies, respectively. Polyclonal anti-H2A.Z antibody was produced by ABclonal^®^ Technology (Wuhan, China). The specificity of polyclonal anti-H2A.Z antibody was checked by western blotting PH-1 and PH-1::H2A.Z-Flag strains (Supplementary Figure S2). The ubiquitination level of FgH2A.Z was immunoprecipitated using polyclonal anti-H2A.Z antibody together with the protein A agarose beads (sc-2001, Santa Cruz, CA, USA), then immunoblotted with a monoclonal anti-ubiquitin antibody (ET1609-21, HuaAn Biotech. Ltd., Hangzhou, China). The samples were also detected with monoclonal anti-GAPDH antibody (EM1101, HuaAn Biotech. Ltd, Hangzhou, China) or monoclonal anti-H3 antibody (M1306-4, HuaAn Biotech. Ltd, Hangzhou, China) as a reference. Each experiment was repeated three times.

### Protein expression and purification

FgUbp8, FgHapX, FgSwc6, FgSreA and FgGrx4 was amplified using PH-1 genomic cDNA, and then cloned into the pET-22a vector (6 × His-tag) for microscale thermophoresis (MST) assays. Briefly, the plasmid was transformed into *E. coli* strain BL21 (DE3), and the cells were cultured in Luria Bertani medium containing 50 μg ml^−1^ ampicillin. Protein expression was induced by adding isopropyl β-d-thiogalactoside (IPTG) to a final concentration of 0.2 mM at 16°C, when the cell culture reached an optimal density at 600 nm of 0.4–0.6 at 37°C in a shaker. After an additional 10 h of culture, bacteria were harvested at 5000 rpm, at 4°C for 10 min. The bacterial pellet was resuspended in lysis buffer containing 200 mM NaCl, 20 mM MES pH 6.5, and 5% glycerol and disrupted by crushing at 800 bar at 4°C for 3 times. The disrupted homogenate was further processed by centrifugation at 12 000 rpm, at 4°C for 60 min and the supernatant was transferred for FPLC system purification. The supernatant containing fusion 6 × His-tag protein was applied on a His trap column (GE, Healthcare), 500 mM imidazole was used as the elution buffer for eluting His-tag fusion protein. Coomassie Brilliant Blue G-250 staining showed the above protein samples resolved by SDS-PAGE (Supplementary Figure S3).

### MST assay

A MST assay was performed using the Monolith NT.115 (NanoTemper Technologies). Target proteins were labeled with RED-tris-NTA (NanoTemper Technologies). Affinity measurements were also performed using MST buffer. Samples were loaded into NT.115 premium capillaries (NanoTemper Technologies). Measurements were performed at 25°C, 80% LED, 20% IR-laser power, and at a constant concentration of 50 nM of labeled protein with increasing concentration of purified protein. Data were analyzed using Nano Temper Analysis Software (NanoTemper Technologies, Germany). Three technical replicates were used for each strain and each experiment was repeated three times independently.

### Bimolecular fluorescence complementation (BiFC) assay

The FgHapX-CYFP and FgHapB-NYFP fusion constructs were generated by cloning the related fragments into pHZ68 and pHZ65 vectors, respectively. FgHapX-CYFP and FgHapB-NYFP constructs were co-transformed into PH-1. In addition, construct pairs FgHapX-CYFP and NYFP, FgHapB-NYFP and CYFP were used as negative controls. Transformants resistant to both hygromycin and zeocin were isolated and confirmed by PCR. Each strain was treated with or without 10 mM FeSO_4_ after culture in CM for 36 h, and then the YFP signals were examined using a Zeiss LSM780 confocal microscope (Gottingen, Niedersachsen, Germany). Using a similar strategy, the interaction of FgHapC-NYFP and FgHapX-CYFP, FgGrx4-NYFP and FgHapX-CYFP, FgGrx4-NYFP and FgSreA-CYFP, with or without iron treatment was determined, respectively. The experiment was conducted three times independently.

### Affinity capture-mass spectrometry analysis

FgSreA was tagged with -GFP and transferred into the *FgSREA* deletion mutant. The resulting transformant was used for protein extraction. After protein extraction, the supernatant was transferred to a sterilized tube. An aliquot of 25 μl of GFP-trap agarose beads (ChromoTek, Martinsried, Germany) was added to capture FgSreA-GFP interacting proteins, following the manufacturer's instructions. After incubation at 4°C overnight, the agarose beads were washed three times with 1000 μl of TBS (20 mM Tris–HCl, 500 mM NaCl, pH 7.5). Proteins bound to the beads were then boiled with 50 μl TBS supplemented with 10 μl 10% SDS. After centrifugation at 5000 *g* for 5 min at 4°C, the supernatant was digested with trypsin, and tryptic peptides were analyzed by Shanghai Applied Protein Technology Co., Ltd. using mass spectrometry. The false discovery rate (FDR) was used to determine relative protein confidence. The peptide-to-spectrum matches (PSMs) were quantified to determine relative protein abundance. Candidate interacting proteins were filtered based on high confidence (≤1% FDR) and reproducible presence across samples (PSM > 5). Two biological replicates were conducted for the strain.

### Isolation of chromatin bound proteins

The isolation of chromatin bound proteins assay was conducted according to a previously reported protocol (50) with some modifications. Briefly, fresh mycelia of each strain were frozen in liquid nitrogen and ground to a fine powder using a mortar and pestle. The resulting powdered mycelia (0.6 g) were mixed with 1 ml of Honda buffer (20 mM HEPES–KOH [pH 7.4], 0.44 M sucrose, 1.25% Ficoll, 2.5% Dextran T40, 10 mM MgCl_2_, 0.5% Triton X-100, 5 mM DTT, 1 mM PMSF and 1% protease inhibitor). The homogenates were then filtered through cheesecloth and then centrifuged at 1500 × *g* for 5 min at 4°C. The pellets were washed one time with 1× PBS (1 mM EDTA). The pellets were then re-suspended in 0.5 ml of glycerol buffer (20 mM Tris–HCl [pH 7.9], 75 mM NaCl, 0.5 mM EDTA, 50% glycerol, 0.85 mM DTT, 0.125 mM PMSF, 1% protease inhibitor and 10 mM b-mercaptoethanol) followed by addition of 0.5 ml of nuclei lysis buffer (10 mM HEPES [pH 7.6], 7.5 mM MgCl_2_, 0.2 mM EDTA, 0.3 M NaCl, 1 M urea, 1 mM DTT, 1% NP-40, 0.5 mM PMSF, 1% protease inhibitor and 10 mM b-mercaptoethanol). The samples were incubated on ice for 2 min followed by centrifugation at 14 000 × g for 2 min at 4°C, the supernatant containing chromatin bound proteins was collected. The experiment was conducted three times independently.

### Chromatin immunoprecipitation (ChIP)-seq and ChIP-qPCR analyses

ChIP was performed as previously described with additional modifications (51). Briefly, fresh mycelia were cross-linked with 1% formaldehyde for 15 min and then stopped with 125 mM glycine. The cultures were ground with liquid nitrogen and resuspended in lysis buffer (250 mM HEPES [pH 7.5], 150 mM NaCl, 1 mM EDTA, 1% Triton, 0.1% deoxycholate, 10 mM DTT) and 1% protease inhibitor. The DNA was sheared into ∼300 bp fragments with 20 pulses of 10 s with 20 s of resting at 35% amplitude (Qsonica*sonicator, Q125, Branson, USA). After centrifugation, the supernatant was diluted with 10× ChIP dilution buffer (1.1% Triton X-100, 1.2 mM EDTA, 16.7 mM Tris–HCl [pH 8.0], 167 mM NaCl). Samples were pre-washed with 20 μl of protein A agarose (sc-2001, Santa Cruz, CA, USA) for 1 h at 4°C. Then immunoprecipitation was performed using a monoclonal anti-H3K27 me3 (39155, Active Motif, Shanghai, China), polyclonal anti-H2A.Z (ABclonal^®^ Technology, Wuhan, China) or monoclonal anti-GFP (ab290, Abcam, Cambridge, UK) antibody together with protein A agarose (Santa Cruz, CA, USA). A mock sample was incubated with anti-IgG antibody (MA1-10406, Invitrogen-Thermo Fisher Scientific, Waltham, MA, USA). DNA was immunoprecipitated with ethanol after washing, eluting, reversing the cross-linking, and digesting with proteinase K.

Further, ChIP-enriched DNA with a monoclonal anti-H3K27 me3 or polyclonal anti-H2A.Z antibody were sequenced on an Illumina NovaSeq 6000. Following adaptor trimming by fastp (52), reads were mapped against the sequence of *F. graminearum* chromosomes using Bowtie2 (53), PCR duplications were removed by Sambamba (54), coverage tracks were generated with the script bamCompare available in deepTools with input DNA as a control (55), peaks were called using MACS2 at *P* value ≤0.005 with input DNA as a control (https://github.com/macs3-project/MACS). The Input was used to eliminate the background noise, and the ChIP signals were calculated as log_2_(IP_RPKM_/Input_RPKM_) for each 10 bp (37). Then KEGG pathway enrichment analysis was implemented using phyper in R package with a corrected *P* value ≤0.01 to identify significantly enriched biological pathways. ChIP-enriched DNA with a monoclonal anti-GFP antibody were used for quantitative PCR analysis using SYBR green I fluorescent dye detection with the relative primers (Supplementary Table S1). The relative enrichment of each gene was determined by quantitative PCR and calculated by normalizing the value of the immunoprecipitated sample with that of the input. ChIP-seq was performed twice independently. Two technical replicates were used for each strain and ChIP-qPCR was repeated three times independently.

### The micrococcal nuclease (MNase)-qPCR assays

The MNase assay was conducted according to a previously reported protocol (56) with some modifications. Briefly, to isolate fungal nuclei, fresh mycelia of each strain were frozen in liquid nitrogen and ground to a fine powder using a mortar and pestle. The resulting powdered mycelia (0.6 g) were mixed with 1 ml of lysis buffer (250 mM sucrose, 25% [v/v] glycerol, 2 mM MgCl_2_, 20 mM KCl, 20 mM Tris–HCl [pH 7.5], 5 mM DTT) and incubated on ice for 5 min with constant stirring. The homogenates were then filtered through cheesecloth and centrifuged at 1500 × *g* for 15 min at 4°C. The pellets were re-suspended in 1 ml nuclei extraction buffer NEB1 (20 mM Tris–HCl [pH 7.5], 0.2% [w/v] Triton X-100, 25% [v/v] glycerol, 2.5 mM MgCl_2_) and centrifuged at 15 000 × *g* for 10 min at 4°C. This step was repeated five times. The resulting pellets were re-suspended in 1 ml NEB2 buffer (20 mM Tris–HCl [pH 7.5], 0.5% [w/v] Triton X-100, 250 mM sucrose, 10 mM MgCl_2_, 5 mM β-mercaptoethanol), layered onto 1 ml of NEB3 buffer (20 mM Tris–HCl [pH 7.5], 0.5% [w/v] TritonX-100, 1.7 M sucrose, 10 mM MgCl_2_, 5 mM β-mercaptoethanol), and centrifuged at 16 000 × *g* for 45 min at 4°C. The final nuclei pellet was suspended in MNase reaction buffer. To conduct MNase digestion, an equal portion of nuclei isolation (160 μl) of each sample was mixed with 320 μl of MNase reaction buffer and treated with 4 μl MNase enzyme (Takara, Beijing, China). The same amount of nuclei isolation without MNase treatment was used as an undigested control. Samples were incubated at 37°C for 8 min, and the reaction was terminated by adding 50 μl of stop buffer (50 μl 10% SDS, and 40 μg proteinase K) at 60°C for 1 h. Each resulting sample was then treated with 1 U RNase (10 μg/μl) at 37°C for 1 h and stored at 4°C overnight. DNA from each sample was extracted using the phenol-chloroform-isoamyl alcohol method and re-suspended in 50 μl water, and 10 μl sample was used to detect the digestion level. The resulting DNA samples were used for quantitative PCR with multiple primer pairs spanning the tested region (Supplementary Table S1). The resulting amplicons, with an average size of 100 bp and 20 bp overlap, were used to analyze nucleosome occupancy as described previously (56). Two technical replicates were used for each strain and each experiment was repeated three times independently.

## RESULTS

### FgHapX and FgSreA co-regulate the adaptation to iron excess of *F*.*graminearum* during infection

**Figure 1.**
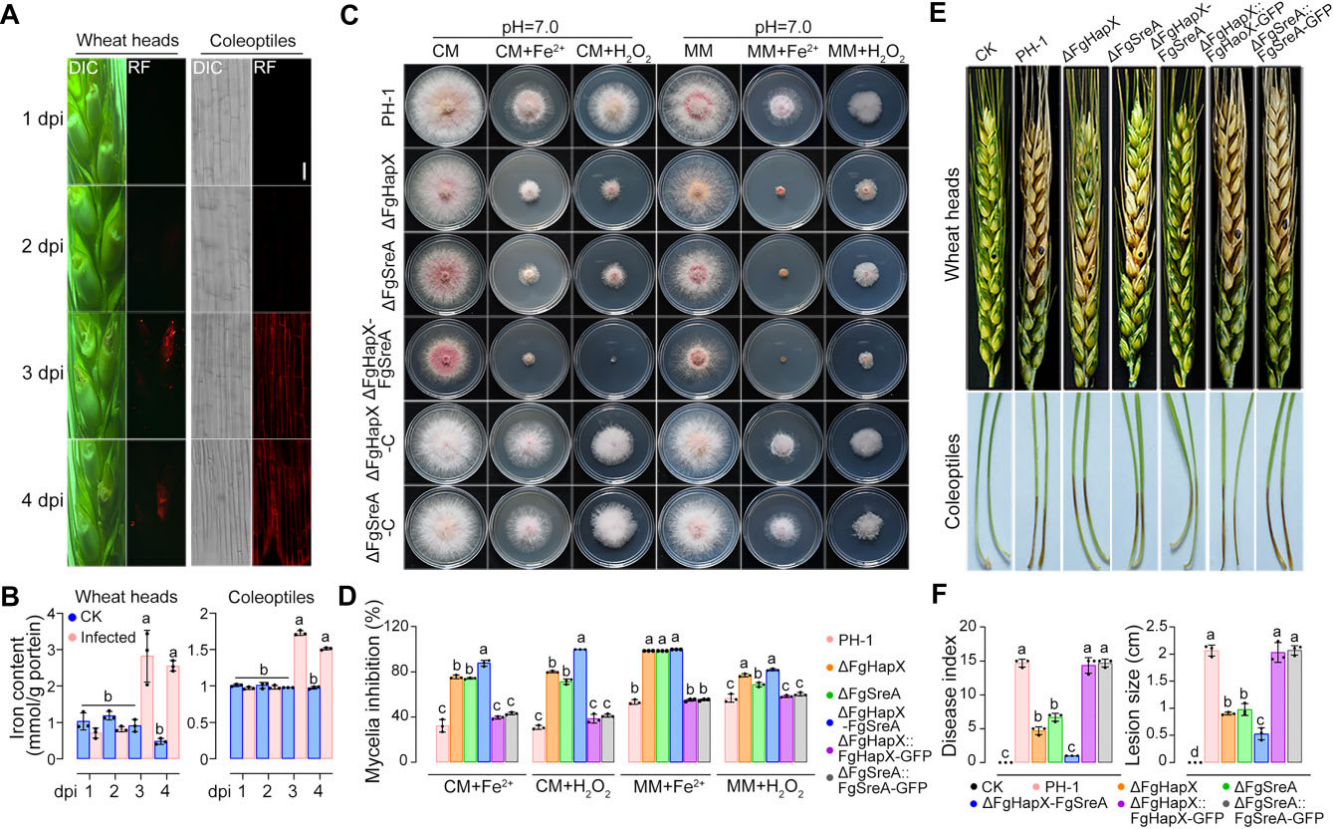
FgHapX and FgSreA are both involved in iron excess adaptation during infection in *F. graminearum*. (A, B) Wheat heads and coleoptiles produced excessive iron during *F. graminearum* infection. Iron content of each spikelet and coleoptile was detected by a laser scanning microscope with 5 μM fluorescent iron-binding dye FeRhoNox-1 (**A**) and iron content of each wheat head was determined by colorimetric ferrozine-based assay (**B**) at the corresponding time point. Dpi: days post inoculation. Sterile water was used as the control (CK). Bar = 50 μm. (C, D) Deletion of FgHapX, FgSreA or FgHapX-SreA led to increased sensitivity to iron excess and hydrogen peroxide (H_2_O_2_). Colony morphology was observed (**C**), and mycelial inhibition was determined (**D**) after growth on complete medium (CM) and minimal medium (MM) with or without 10 mM Fe_2_SO_4_ or 0.05% H_2_O_2_ for 3 days. (E, F) Lack of FgHapX, FgSreA or FgHapX-SreA caused significantly reduced virulence on wheat heads and coleoptiles. Representative images of wheat heads were photographed (**E**), and disease index was calculated (**F**) at 15 dpi. The inoculated site on each wheat head was labeled with a black dot. Representative images of coleoptiles were photographed (E), and lesion sizes were measured (F) at 4 dpi. Sterile water was used as the control (CK).

Plant hosts often drive iron starvation or excess to prevent pathogen infection (6,57). More recently, *F. graminearum* was reported to be challenged by both iron excess and alkalinization during infection (37), we therefore monitored the iron content and alkalinization at infection sites from 1 to 4 or 7 dpi. Microscopic examination using the fluorescent iron-binding dye FeRhoNox-1 (22) showed that the content of iron in the infected wheat heads and coleoptiles increased at 3 and 4 dpi (Figure 1A). Accordingly, quantitative analysis based on the colorimetric ferrozine-based assay (22) showed that the iron content of wheat heads and coleoptiles elevated at 3 and 4 dpi (Figure 1B). We adapted a bioassay in plates containing different pH indicators and observed significant extracellular alkalinization surrounding the wheat coleoptiles and seedling leaves starting at 7 dpi (Supplementary Figure S4A). Moreover, alkalinization was also detected in the apoplastic fluids of coleoptiles and seedling leaves at 6 dpi (Supplementary Figure S4B). These results demonstrated that *F. graminearum* is confronted with iron excess before alkalinization during the infection of wheat (cultivar Jimai22).

To explore how *F. graminearum* counteracts iron excess before alkalinization during infection, we determined the sensitivity to iron excess, H_2_O_2_, and virulence of iron-responsive transcription factors FgHapX and FgSreA, whose homologs have been reported to be responsible for iron resistance in fungi (19,22–24,58,59). The mutants ΔFgHapX and ΔFgSreA displayed increased sensitivity to iron excess under pH 7.0 (Figure 1C, D). Moreover, compared with the single mutants, the double mutant ΔFgHapX-FgSreA exhibited a more severe growth defect under iron excess treatment (Figure 1C, D). Given that iron excess in cells leads to the formation of hydroxyl radicals via the Fenton reaction (60), we determined the sensitivity to H_2_O_2_ and found that all three mutants showed elevated sensitivity to H_2_O_2_ (Figure 1C, D). The virulence of each mutant was evaluated on flowering wheat heads and coleoptiles. Deletion of FgHapX or FgSreA resulted in significantly reduced virulence, whereas the deletion of both led to restricted scab symptoms only in the inoculated sites (Figure 1E, F). On the other hand, the complemented strains ΔFgHapX::FgHapX-GFP and ΔFgSreA::FgSreA-GFP displayed similar sensitivity to iron excess and H_2_O_2,_ as the wild type, and caused typical FHB symptoms on wheat heads and coleoptiles under the same conditions (Figure 1C–F). These results indicate that FgHapX and FgSreA can jointly regulate the adaptation to iron excess during *F. graminearum* infection.

### During iron excess, FgHapX activates iron storage and FgSreA represses iron acquisition

**Figure 2.**
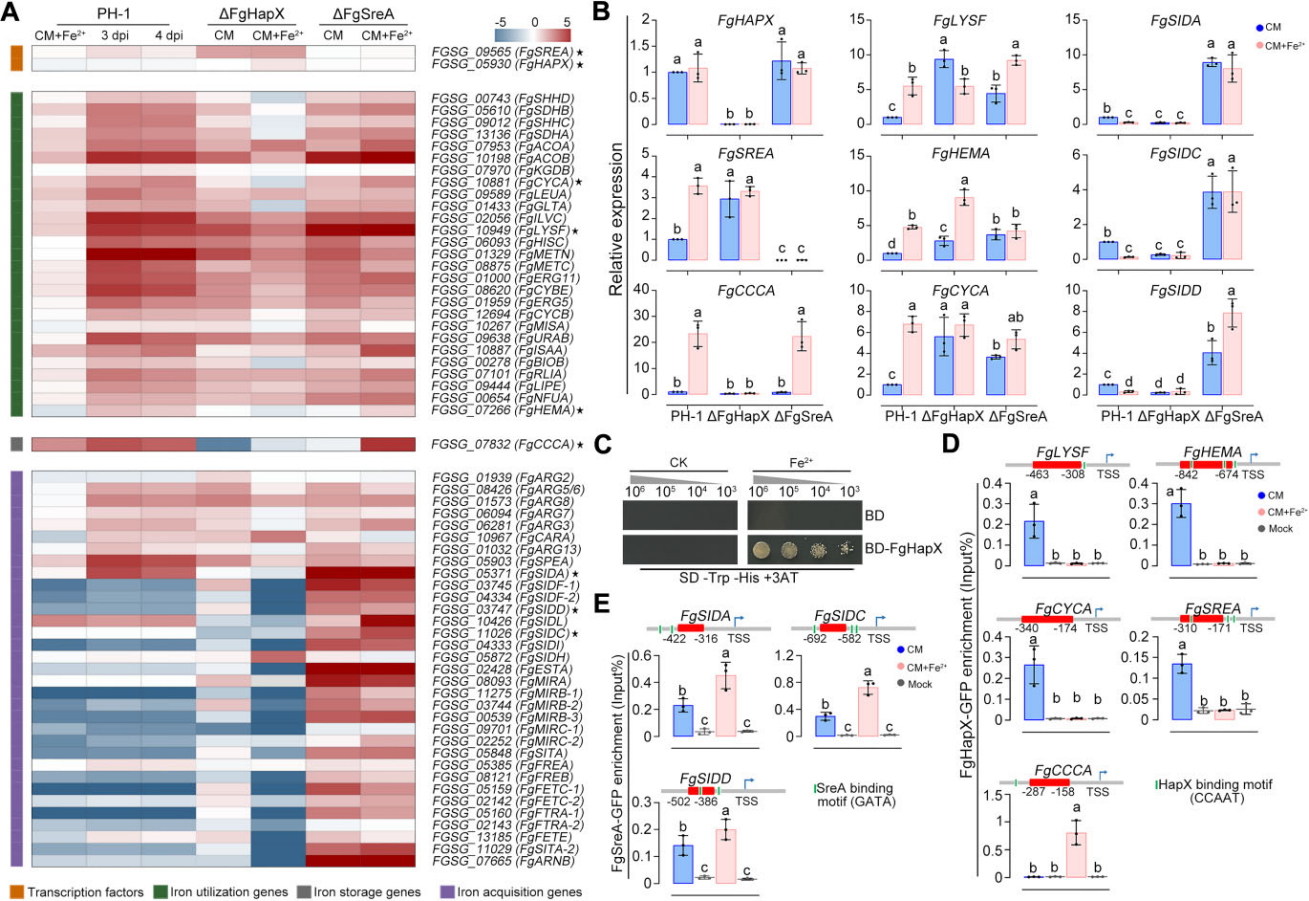
FgHapX and FgSreA co-regulate the transcription of iron homeostasis genes upon iron excess. (**A**) Heatmap of differentially expressed iron homeostasis genes in the wild type (PH-1) at 3 and 4 days post inoculation (dpi), or in PH-1, ΔFgHapX and ΔFgSreA with or without 10 mM Fe_2_SO_4_. The expression level of each gene in PH-1 cultivated with CM was set to 1. Red color indicates relatively high expression, and blue color indicates relatively low expression. Genes marked with stars were used for qRT-PCR assays. (**B**) Transcription of iron homeostasis genes in PH-1, ΔFgHapX and ΔFgSreA, with or without iron excess treatment in qRT-PCR assays. The expression level of each gene in PH-1 in CM without iron excess treatment was set to 1 and the *FgACTIN* gene was used as the internal control for normalization. (**C**) FgHapX is able to activate transcription upon iron excess in a yeast transcriptional activation activity assay. Serial dilutions of yeast cells (cells/ml) transferred with the plasmid pGBKT7 (BD)-FgHapX or pGBKT7 were assayed for growth on SD-Trp-His + 3AT plates with or without 10 mM Fe^2+^ (CK). (D, E) The enrichment of FgHapX-GFP at the promoters of iron utilization and storage genes and *FgSREA* (**D**), and the occupancy of FgSreA-GFP at the promoters of iron acquisition genes (**E**) in ChIP-qPCR assays. The input-DNA and ChIP-DNA samples were quantified by quantitative PCR assays with corresponding primer pairs (Supplementary Table S1). The enrichment localization for each gene was shown in lower right panels in figure 2D and E. ChIP signals are shown as the percentages of input and mock-DNA incubated with anti-IgG antibody as a control. TSS: transcription start site. In (B, D and E), mean and standard deviation were estimated with data from three independent biological replicates (marked with dots in different color, *n* = 3). Different letters indicate statistically significant differences according to the one-way ANOVA test (*P*< 0.05).

RNA-seq assays were performed for the wild type at 3 dpi and 4 dpi during plant infection or in CM culture with or without iron excess treatment to explore the transcriptional changes of iron homeostasis genes during infection and upon iron excess. Based on previous studies (20,22), a total of 63 genes including transcription factors *FgHAPX* and *FgSREA*, 27 iron utilization genes, one iron storage gene *FgCCCA*, and 33 iron acquisition genes were analyzed. Relative to CM culture without treatment, transcription profiling analyses showed that *FgSREA*, *FgCCCA* and all iron utilization genes were induced, while most (20/33) of the iron acquisition genes were suppressed at 3 and 4 dpi, as also found under iron excess treatment (Figure 2A). These data reveal that the transcription of iron homeostasis genes at 3 and 4 dpi is similar to that upon iron excess.

To better illustrate the functions of FgHapX and FgSreA in the regulation of iron resistance during infection, we conducted RNA-seq assays for ΔFgHapX, and ΔFgSreA using iron excess treatment to mimic plant infection conditions. *FgHAPX* transcription was not affected by iron excess treatment or deletion of *FgSREA* (Figure 2A, B). Ten iron utilization genes including *FgLYSF*, *FgHEMA*, *FgCYCA* and *FgSREA* were induced upon iron excess (Figure 2A, B). The transcription of iron utilization genes and *FgSREA* was repressed by FgHapX (Figure 2A, B) particularly without iron treatment, which is consistent with the findings of a previous study (22). The iron storage gene *FgCCCA* was induced by iron excess, and its transcription was significantly attenuated in ΔFgHapX with or without iron excess treatment (Figure 2A, B), suggesting transcriptional activation by FgHapX. Accordingly, a yeast transcriptional activation activity assay showed that FgHapX could activate transcription under iron excess (Figure 2C), although *S. cerevisiae* lacks a HapX ortholog (61). Furthermore, ChIP-qPCR assays with the complemented strain ΔFgHapX::FgHapX-GFP were performed to detect the enrichment of FgHapX in the promoter regions containing HapX-binding motifs (CSAATN_12_RWT) (27). ChIP-qPCR analyses revealed that FgHapX was enriched at the promoters of iron utilization genes and *FgSREA*, but not at the *FgCCCA* promoter without iron excess treatment (Figure 2D). In contrast, FgHapX only occupied the *FgCCCA* promoter, but not the promoters of iron utilization genes and *FgSREA*, under iron excess conditions (Figure 2D). These results indicate that FgHapX derepresses iron utilization genes and *FgSREA* via dissociation from their promoters, while it occupies the *FgCCCA* promoter to activate its transcription upon iron excess. In addition, FgSreA deficiency also led to the upregulation of iron utilization genes with or without iron excess treatment (Figure 2A, B), which might be due to iron accumulation in ΔFgSreA. However, deletion of FgSreA did not affect *FgCCCA* transcription without iron excess (Figure 2A, B); most likely because the cellular iron accumulation was not as high as under iron excess.

In *F. graminearum*, FgSreA represses the transcription of iron acquisition genes (22). In this study, 22 iron acquisition genes including *FgSIDA, FgSIDC*, and *FgSIDD* were downregulated upon iron excess treatment and were inhibited by FgSreA (Figure 2A, B). Further, ChIP-qPCR analyses with the complemented strain ΔFgSreA::FgSreA-GFP revealed that FgSreA occupied the promoter regions carrying SreA-binding motifs (ATCWGATA) (24). Moreover, such enrichments were increased by iron excess treatment (Figure 2D). The loss of FgHapX led to downregulation of iron acquisition genes with or without iron excess, which resulted from the upregulation of *FgSREA* in ΔFgHapX (Figure 2A, B). Taken together, these results indicate that FgHapX induces the iron storage gene as well as derepresses *FgSREA* and iron utilization genes, while FgSreA inhibits iron acquisition genes during response to iron excess in *F. graminearum*.

In addition, HapX of *A. fumigatus* executes all functions via physical interaction with the heterotrimer CCAAT-binding complex (CBC), which contains three components: HapB, HapC, and HapE (27). Therefore, we determined the effect of CBC on FgHapX functions. Since we were unable to delete FgHapB, FgHapC or FgHapE after obtaining >300 ectopic transformants for each gene from four independent transformation experiments, we knocked down FgHapB (*FgHapB^KD^*) by replacing the FgHapB promoter with Pzear (48) (Supplementary Figure S1). Similar to ΔFgHapX (22), *FgHapB^KD^* displayed increased sensitivity to iron excess and unchanged intracellular iron content (Supplementary Figure S5A–D). The qRT-PCR assays showed that knockdown of FgHapB upregulated the expression of iron utilization genes and *FgSREA* particularly without iron treatment, and compromised the induced expression of *FgCCCA* upon iron excess (Supplementary Figure S5E), reminiscent of that in FgHapX. Moreover, the Y2H and BiFC assays showed that FgHapX interacts with both FgHapB and FgHapC, but not with FgHapE (Supplementary Figure S5F, G). These results indicate that CBC may assist FgHapX to tune iron homeostasis.

### FgHapX activates *FgCCCA* transcription via enhancing H2B deub1 upon iron excess

**Figure 3.**
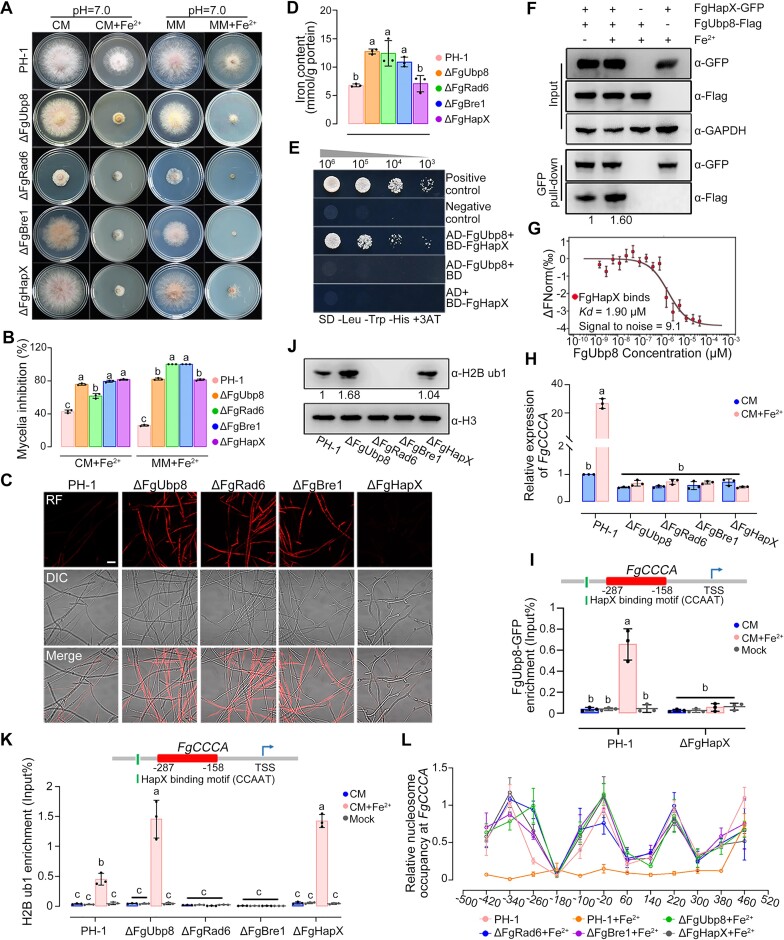
FgHapX activates *FgCCCA* transcription via promoting H2B deub1 upon iron excess. (A, B) Lack of ubiquitin protease FgUbp8, H2B ub1 conjugated enzyme FgRad6, H2B ub1 ligase FgBre1 or FgHapX led to increased sensitivity to iron excess. Colony morphology was observed (**A**), and mycelial inhibition was determined (**B**) after growth on CM with or without 10 mM Fe_2_SO_4_ for 3 days. (C, D) The ΔFgUbp8, ΔFgRad6 and ΔFgBre1 displayed increased iron content, compared to the wild type and ΔFgHapX. Iron content of each strain was determined by a laser scanning microscope with 5 μM fluorescent iron-binding dye FeRhoNox-1 (**C**) or colorimetric ferrozine-based assay (**D**) after culture in CM for 36 h. Bar = 20 μm. (**E**) FgHapX interacts with FgUbp8 in yeast two-hybrid (Y2H) assays. Serial dilutions of yeast cells (cells/ml) transferred with the bait and prey constructs were assayed for growth on SD-Leu-Trp-His + 3AT plates. A pair of plasmids pGBKT7 (BD)-53 and pGADT7 (AD)-T was used as a positive control. A pair of plasmids pGBKT7-Lam and pGADT7-T was used as a negative control. (**F**) Co-immunoprecipitation (Co-IP) assays showed FgHapX interacts with FgUbp8 and the interaction was elevated by iron excess treatment. The protein samples detected with anti-GAPDH antibody were used as a reference. The intensities of the western blotting bands were quantified with the program ImageJ. Values on the bars are the intensity of detected protein band relative to that of GAPDH band. (**G**) Microscale thermophoresis (MST) assay revealed the interaction of FgHapX and FgUbp8. Labeled FgHapX protein (50 nM) was incubated with a serial concentration of FgUbp8 protein and detected by Monolith NT.115 [NT.115Pico]. An MST time of 1.5 s was used for analysis, and a *K*_d_ value was derived for this interaction. Three independent repeats were performed and data represent mean ± SE from three independent replicates (*n* = 3). (**H**) Deletion of FgUbp8, FgRad6, FgBre1 or FgHapX impeded the up-regulation of *FgCCCA* under iron excess. The expression level of *FgCCCA* in the wild type (PH-1) in CM without iron excess treatment was set to 1 and the *FgACTIN* gene was used as the internal control for normalization. (**I**) ChIP-qPCR assays revealed that iron excess treatment increased FgUbp8-GFP enrichment at *FgCCCA* promoter, and the increased FgUbp8 enrichment relied on FgHapX. TSS: transcription start site. (**J**) Western blot assays showed that FgUbp8 modulated H2B deub1 level, FgRad6 and FgBre1 regulated H2B ub1 level. H2B ub1 level was detected with the anti-H2B ub1 antibody. H3 level detected with anti-H3 antibody was conducted as the protein loading reference. The intensities of the western blotting bands were quantified with the program ImageJ. Values on the bars are the intensity of detected protein band relative to that of H3 band. (**K**) ChIP-qPCR assays revealed that iron excess treatment increased H2B ub1 enrichment at *FgCCCA* promoter, and lack of FgRad6 or FgBre1 caused the loss of H2B ub1 enrichment, deletion of FgHapX or FgUbp8 led to excessive H2B ub1 enrichment with or without iron excess treatment. TSS: transcription start site. (**L**) MNase-qPCR assays showed that the H2B ub1/deub1 cycle regulated nucleosomal positioning rearrangement in *FgCCCA* promoter. *FgCCCA* upstream was packaged in a positioned array of nucleosomes in CM without iron excess treatment, while the positioned nucleosome array was lost allowing for exposure of a nucleosome-free region when treated by iron excess. However, the positioned nucleosome array persisted in ΔFgUlp8, ΔFgRad6, ΔFgBre1 and ΔFgHapX in CM with iron excess. In (J, K), the input-DNA and ChIP-DNA samples were quantified by quantitative PCR assays with corresponding primer pairs (Supplementary Table S1). ChIP signals are shown as the percentages of input and mock-DNA incubated with anti-IgG antibody as a control. In (B, D, H, I and K), mean and standard deviation were estimated with data from three independent biological replicates (marked with black or gray dots, *n* = 3). Different letters indicate statistically significant differences according to the one-way ANOVA test (*P*< 0.05).

To explore the FgHapX-mediated transcriptional activation mechanism of the iron storage gene *FgCCCA*, we screened a *F. graminearum* cDNA library constructed under iron excess treatment using the Y2H approach and identified 45 potential FgHapX-interacting proteins (Supplementary Table S2). Compared with the interactors of FgHapX obtained using the cDNA library constructed under normal conditions (22), 21 new potential interacting proteins attracted our attention (marked in Supplementary Table S2). We scanned for transcription-related proteins such as histone modifiers, transcription factors or transcriptional elongation factors among the 21 candidates, and then focused on the putative H2B ub1 deubiquitinating enzyme FgUbp8 (FGSG_07958). Notably, the FgUbp8 deletion mutant ΔFgUbp8 showed increased sensitivity to iron excess and elevated cytosolic iron content by using the fluorescent iron-binding dye FeRhoNox-1 and colorimetric ferrozine-based assay (22) (Figure 3A–D). Furthermore, the association between FgHapX and FgUbp8 was confirmed by Y2H and Co-IP (Figure 3E, F), and the interaction between FgHapX and FgUbp8 was enhanced by iron excess treatment in the Co-IP assays (Figure 3F). Accordingly, an MST generated dose response binding affinity curve showed that FgHapX bound FgUbp8 with a high signal to noise ratio (Figure 3G). Moreover, deletion of *FgUBP8* dramatically decreased the expression levels of *FgCCCA* treated with iron excess (Figure 3H). ChIP-qPCR analyses revealed that the occupancy of FgUbp8 at the *FgCCCA* promoter was increased upon iron excess using the same primer pair as for detecting FgHapX enrichment (Supplementary Table S1). Moreover, FgUbp8 enrichment was dependent on the presence of FgHapX (Figure 3I). In comparison, ChIP-qPCR analyses showed that FgUbp8 was not enriched at the promoters of iron utilization genes and *FgSREA*, which are repressed by FgHapX without iron treatment (Supplementary Figure S6). Given that Ubp8 is the catalytic subunit of the SAGA deubiquitinase (DUB) module in budding yeast and humans (62–64), we determined the histone H2B ubiquitination (H2B ub1) levels of the ΔFgUbp8 and ΔFgSgf73 (another component of DUB module). As shown in Figure 3J and Supplementary Figure S7, only ΔFgUbp8 showed significantly higher H2B ub1 level compared to the wild type. And as expected, lack of FgUbp8 or FgHapX increased H2B ub1 enrichment at the *FgCCCA* promoter with or without iron excess treatment (Figure 3K). These results indicate that FgHapX activates *FgCCCA* transcription by promoting H2B deub1 at its promoter upon iron excess in *F. graminearum*.

In budding yeast and *Arabidopsis*, both histone H2B ub1 and deub1 are related to transcriptional activation (65–68). We therefore analyzed whether H2B ub1 is also involved in the transcriptional activation of *FgCCCA*. In *F. graminearum*, FgRad6 and FgBre1 have been identified as H2B ub1 conjugated and ligase enzymes, respectively (45). We found that like ΔFgUbp8, the deletion mutants ΔFgRad6 and ΔFgBre1 displayed elevated sensitivity to iron excess, an increased cytosolic iron content, and reduced expression of *FgCCCA* upon iron excess (Figure 3A–D, H). Further, determination of H2B ub1 enrichment at the *FgCCCA* promoter using the above primers revealed that deletion of FgRad6 or FgBre1 caused the loss of H2B ub1 enrichment induced by iron excess (Figure 3K), indicating that lack of H2B ub1 as well as overload of H2B ub1, impeded the induced expression of *FgCCCA* by iron excess (Figure 3H, K). To further confirm the function in transcription activation of H2B ub1/deub1 cycle, we analyzed chromatin tightness at *FgCCCA* promoter using MNase digestion experiments. The DNAs from different strains separated on agarose gels displayed similar MNase digestion levels (Supplementary Figure S8). The DNA of *FgCCCA* upstream was packaged in a positioned array of nucleosomes without iron excess treatment, while the positioned nucleosome array was reduced after treatment with iron excess (Figure 3L). However, the positioned nucleosome array persisted in ΔFgHapX, ΔFgUbp8, ΔFgRad6 and ΔFgBre1 under iron excess treatment (Figure 3L), indicating that FgHapX- and FgUbp8-mediated H2B deub1 and FgRad6- and FgBre1-mediated H2B ub1 remodel chromatin structure in the promoter of *FgCCCA*. These results indicate that H2B ub1/deub1 cycle participates in *FgCCCA* transcription activation upon iron excess in *F. graminearum*.

### FgSreA inhibits transcription of iron acquisition genes via facilitating the deposition of H2A.Z

**Figure 4.**
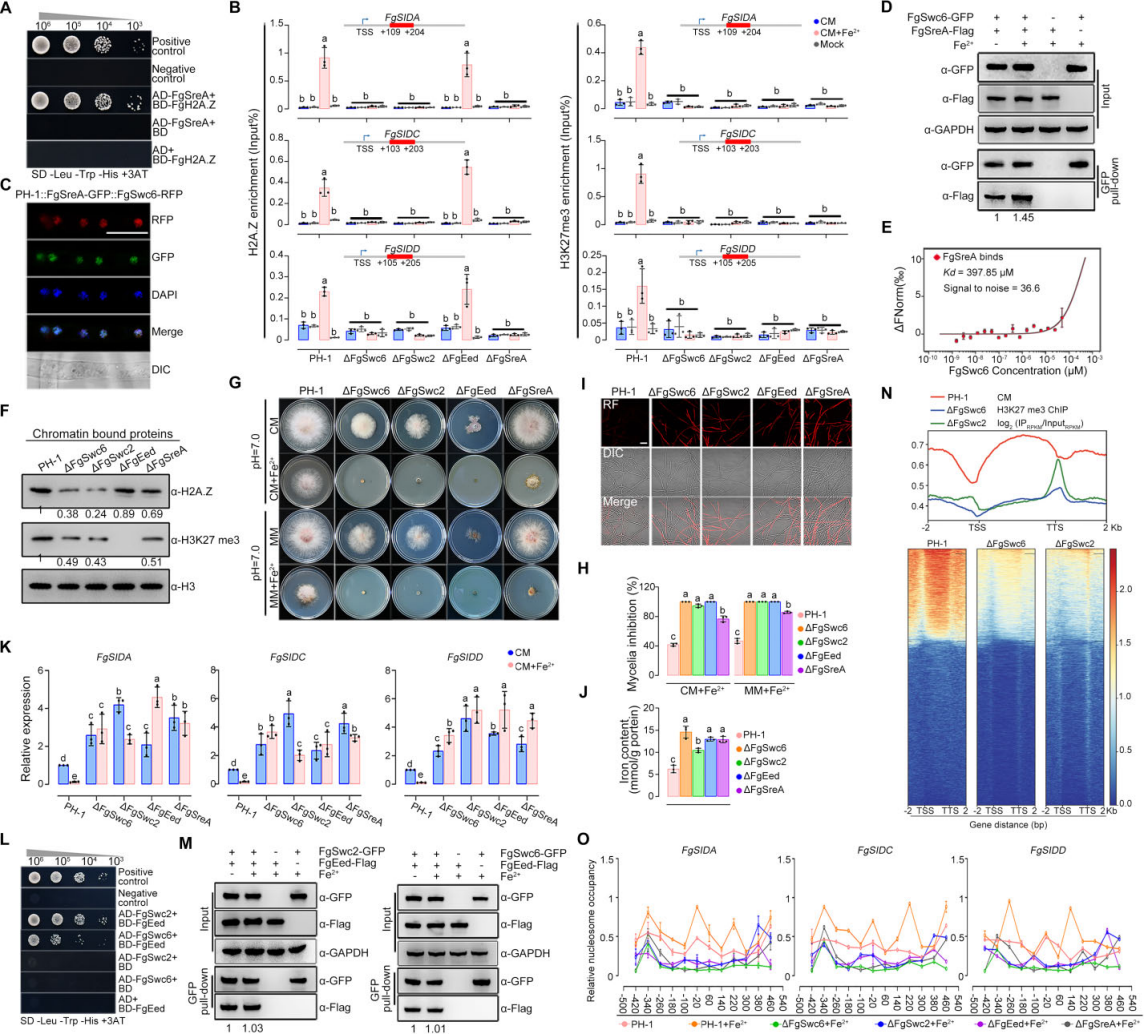
FgSreA represses transcription via enhancing the deposition of H2A.Z and H3K27 me3 on iron acquisition genes. (**A**) FgSreA interacts with FgH2A.Z in Y2H assays. (**B**) H2A.Z and H3K27 me3 were enriched at the first nucleosome after the transcription start site (+1 nucleosome) of iron acquisition genes in ChIP-qPCR assays, moreover these two enrichments were increased by iron excess treatment, and dependent on the presence of FgSreA, FgSwc6 and FgSwc2. The input-DNA and ChIP-DNA samples were quantified by quantitative PCR assays with corresponding primer pairs (Supplementary Table S1). ChIP signals are shown as the percentages of input and mock-DNA incubated with anti-IgG antibody as a control. TSS: transcription start site. (**C**) FgSreA-GFP and FgSwc6-RFP co-localized in the nucleus in CM with iron excess treatment. Bar = 10 μm. (**D**) FgSreA interacts with FgSwc6 and the interaction was elevated by iron excess treatment in Co-IP assays. (**E**) Microscale thermophoresis (MST) assay verified the interaction of FgSreA and FgSwc6. Labeled FgSreA protein (50 nM) was incubated with a serial concentration of FgSwc6 protein and detected by Monolith NT.115 [NT.115Pico]. An MST time of 1.5 s was used for analysis, and a *K*_d_ value was derived for this interaction. Three independent repeats were performed and data represent mean ± SE from three independent replicates (*n* = 3). (**F**) Western blot assays of chromatin bound proteins showed that FgEed is responsible for H3K27 me3, and FgSwc6 and FgSwc2 are involved in the regulation of H2A.Z and H3K27 me3. H2A.Z and H3K27 me3 were detected with the anti-H2A.Z and anti-H3K27 me3 antibodies. H3 level detected with anti-H3 antibody was conducted as the protein loading reference. (G, H) Deletion of the SWR1 complex subunit FgSwc6 or FgSwc2, the PRC2 complex subunit FgEed or FgSreA resulted in increased sensitivity to iron excess. Colony morphology was observed (**G**), and mycelial inhibition was determined (**H**) after growth on CM with or without 10 mM Fe_2_SO_4_ for 3 days. (**I, J**) The ΔFgSwc6, ΔFgSwc2, ΔFgEed and ΔFgSreA all presented increased iron content compared to the wild type. Iron content of each strain was determined by a laser scanning microscope with 5 μM fluorescent iron-binding dye FeRhoNox-1 (I) or colorimetric ferrozine-based assay (**J**) after culture in CM for 36 h. Bar = 20 μm. Fluorescence intensity was further evaluated by line-scan graph analysis, and the horizontal axis indicates the distance. (**K**) Deletion of FgSwc6, FgSwc2, FgEed or FgSreA caused the up-regulation of iron acquisition genes with or without iron excess treatment. The expression level of each iron acquisition gene in the wild type (PH-1) in CM without iron excess treatment was set to 1 and the *FgACTIN* gene was used as the internal control for normalization. (**L**) FgSwc6 and FgSwc2 both interact with FgEed in Y2H assays. (**M**) FgSwc2 and FgSwc6 interacts with FgEed in Co-IP assays. (**N**) Scatterplots and heat maps for distributions of H3K27 me3 signals in PH-1, ΔFgSwc6 and ΔFgSwc2. The H3K27 me3 signals expand from 2 kb upstream to 2 kb downstream. TSS: transcription start site, TTS: transcription termination site. (**O**) MNase-qPCR assays showed that H3K27 me3 level regulated nucleosomal positioning rearrangement in +1 nucleosome of iron acquisition genes. The +1 nucleosomes of iron acquisition genes were packaged in a positioned array of nucleosomes in CM with iron excess treatment. However, the positioned nucleosome array was lost in ΔFgSwc6, ΔFgSwc2, ΔFgEed and ΔFgSreA in CM with iron excess. In (A, L), serial dilutions of yeast cells (cells/ml) transferred with the bait and prey constructs were assayed for growth on SD-Leu-Trp-His + 3AT plates. A pair of plasmids pGBKT7 (BD)-53 and pGADT7 (AD)-T was used as a positive control. A pair of plasmids pGBKT7-Lam and pGADT7-T was used as a negative control. In (D, F and M), The intensities of the western blotting bands were quantified with the program ImageJ. Values on the bars are the intensity of detected protein band relative to that of GAPDH or H3 band. In (B, H, J and K), mean and standard deviation were estimated with data from three independent biological replicates (marked with black or gray dots, *n* = 3). Different letters indicate statistically significant differences according to the one-way ANOVA test (*P*< 0.05).

FgSreA inhibited the transcription of iron acquisition genes with or without iron excess treatment (Figure 2A, B). To explore the transcriptional repression mechanism, we searched for proteins interacting with FgSreA. The STRING database 11.5 (https://string-db.org) predicted 6 potential interacting proteins, i.e. histone H3, histone variant H2A.Z, SWR1 complex subunits Swr1, Arp6, Rvb2 and Swc4 (Supplementary Figure S9A). Y2H assays were performed to test the interaction of the homologs of these proteins with FgSreA. As shown in Figure 4A and Supplementary Figure S9B, FgSreA interacted only with FgH2A.Z, but not with the other proteins, suggesting that H2A.Z may be involved in the transcriptional repression of iron acquisition. H2A.Z deposition and eviction have been reported to participate in both transcriptional activation and repression in eukaryotic organisms (69). Remarkably, FgH2A.Z could not be deleted or knocked down after obtaining more than 300 ectopic transformants or 200 transformants with unchanged expression levels, which indicates its essentiality as reported previously (70). In a next step, we determined the H2A.Z occupancy near the transcription start site based on previous studies on H2A.Z distribution (71–73). ChIP-qPCR assays revealed that H2A.Z was enriched at the first nucleosome after the transcription start site (+1 nucleosome) of iron acquisition genes *FgSIDA*, *FgSIDC* and *FgSIDD* upon iron excess using anti-H2A.Z antibody (Figure 4B and Supplementary Figure S10), indicating that H2A.Z deposition is associated with the transcriptional repression of iron acquisition gene.

The SWI/SNF2 Related 1 (SWR1) complex contains 14 subunits and is responsible for specifically replacing the H2A-H2B dimer with the H2A.Z-H2B dimer in a stepwise ATP-required reaction in budding yeast (74,75). Given that FgSreA does not directly bind to all the components of SWR1 in Y2H assays (Supplementary Figure S9B), we tried to explore the interaction of FgSreA with the SWR1 by an affinity capture assay. Briefly, FgSreA fused with GFP was produced in ΔFgSreA. The proteins purified using the GFP antibody were further analyzed by mass spectrometry. In addition, a strain transformed with GFP alone was used as a negative control. As shown in Supplementary Table S3, FgSreA may interact with FgSwc6 (FGSG_11916), a homolog of *S. cerevisiae* Swc6, which is required for SWR1 complex binding to the canonical nucleosome substrate in yeast (74). Microscopic observation showed that both FgSreA and FgSwc6 co-localized in the nucleus under iron excess (Figure 4C). Furthermore, the interaction between FgSreA and FgSwc6 was confirmed by Co-IP assays (Figure 4D). Moreover, the interaction between FgSreA and FgSwc6 was enhanced under iron excess (Figure 4D). Accordingly, an MST generated dose response binding affinity curve revealed that FgSreA bound FgSwc6 with a high signal to noise ratio (Figure 4E), indicating that FgSreA interacts with FgSwc6. As expected, lack of FgSwc6 caused reduced H2A.Z level in western blotting analyses with chromatin bound proteins (Figure 4F), suggesting its role in H2A.Z deposition. Phenotypic characterization revealed that the ΔFgSwc6 mutant displayed increased sensitivity to iron excess and accumulated more intracellular iron than the wild type (Figure 4G–J), similar to the cases in ΔFgSreA. Accordingly, qRT-PCR assays showed that expression of iron acquisition genes was upregulated significantly in ΔFgSwc6 and ΔFgSreA, with or without iron excess treatment (Figure 4K). Furthermore, ChIP-qPCR assays revealed that the H2A.Z occupancy at the +1 nucleosome of iron acquisition genes during iron excess depends on both FgSwc6 and FgSreA (Figure 4B).

To confirm that the SWR1 complex-mediated H2A.Z deposition functions in transcription inhibition of iron acquisition genes, we obtained the mutants of another SWR1 complex subunit 2, FgSwc2 (FGSG_02520) that is a widely conserved H2AZ-binding module and essential for histone exchange (Supplementary Figure S1A, B) (74). Similar to ΔFgSwc6, ΔFgSwc2 displayed reduced H2A.Z level, increased sensitivity to iron excess and elevated intracellular iron accumulation (Figure 4F-J). Accordingly, lack of FgSwc2 prevented transcriptional inhibition of iron acquisition genes and H2A.Z enrichment at the +1 nucleosome of iron acquisition genes upon iron excess (Figure 4B). Taken together, these results indicate that FgSreA recruits H2A.Z occupancy at iron acquisition genes by interacting with the SWR1 complex under iron excess conditions.

### H2A.Z deposition enhances H3K27 me3 in the transcriptional repression of iron acquisition genes

Recently, H2A.Z monoubiquitination was reported to play a vital role in transcriptional repression (76). We therefore detected the ubiquitination level of H2A.Z, but did not detect its monoubiquitination in *F. graminearum* with or without iron excess treatment (Supplementary Figure S11). To explore the mechanism for repression of iron acquisition genes regulated by H2A.Z deposition in more detail, the potential interacting proteins of FgH2A.Z were predicted using the STRING database 11.5 (https://string-db.org). Besides the SWR1 complex, polycomb repressive complex-2 (PRC2) that catalyzes H3K27 me3, a widespread repressive epigenetic modification (77–79), was proposed to interact with H2A.Z (Supplementary Figure S9C). We therefore performed Y2H assays to test the interaction of three components of PRC2 (FgKmt6, FgEed and FgSuz12) with FgH2A.Z, FgSwc6, FgSwc2 and other SWR1 complex subunits (80). As shown in Figure 4L and Supplementary Figure S9D, both FgSwc6 and FgSwc2 interact with FgEed, but not with FgKmt6 and FgSuz12, and FgH2A.Z does not bind to them in Y2H assays. Further, Co-IP assays verified the interactions of FgSwc6 and FgSwc2 with FgEed (Figure 4M), suggesting that the SWR1 complex is associated with the PRC2 in *F. graminearum*. It was interesting to determine whether H2A.Z deposition represses the transcription of iron acquisition genes via recruiting H3K27 me3. Western blotting assays revealed that the H3K27 me3 level was undetected in ΔFgEed, and significantly reduced ΔFgSwc2 and ΔFgSwc6 compared with the wild type (Figure 4F). Accordingly, ChIP-seq analyses showed that the enrichment of H3K27 me3 was reduced in ΔFgSwc6 and ΔFgSwc2 compared with the wild type (Figure 4N; Supplementary Figure S12A). And ChIP-qPCR assays revealed that H3K27 me3 was enriched at the + 1 nucleosome of iron acquisition genes and such enrichment was increased by iron excess treatment (Figure 4B), similar to H2A.Z. Moreover, the enrichment of H3K27 me3 was dependent not only on FgEed but also on FgSwc6, FgSwc2 and FgSreA (Figure 4B), suggesting that H2A.Z deposition promotes PRC2-mediated H3K27 me3. In addition, ΔFgEed mutant displayed increased sensitivity to iron excess and elevated iron accumulation (Figure 4G–J). Moreover, qRT-PCR assays demonstrated that the expression of iron acquisition genes was significantly upregulated in ΔFgEed with or without high iron stress (Figure 4K). Together, these results indicate that H2A.Z deposition facilitates H3K27 me3 and both are involved in transcriptional repression of iron acquisition genes upon iron excess.

Since H3K27 me3 is known as a marker of transcriptional repression in eukaryotic organisms (35,79), we thus detected chromatin tightness in the iron acquisition genes using MNase digestion experiments. The DNAs of different strains exhibited similar MNase digestion levels (Supplementary Figure S8). The DNA of iron acquisition genes + 1 nucleosome regions was packaged in a positioned array of nucleosomes under iron excess conditions (Figure 4O). However, the positioned nucleosome array was lost in ΔFgSreA, ΔFgSwc6, ΔFgSwc2 and ΔFgEed upon iron excess (Figure 4O). These results indicate that FgSreA recruits H2A.Z and H3K27 me3 to decrease nucleosome accessibility upon iron excess, subsequently repressing the transcription of iron acquisition genes.

**Figure 5.**
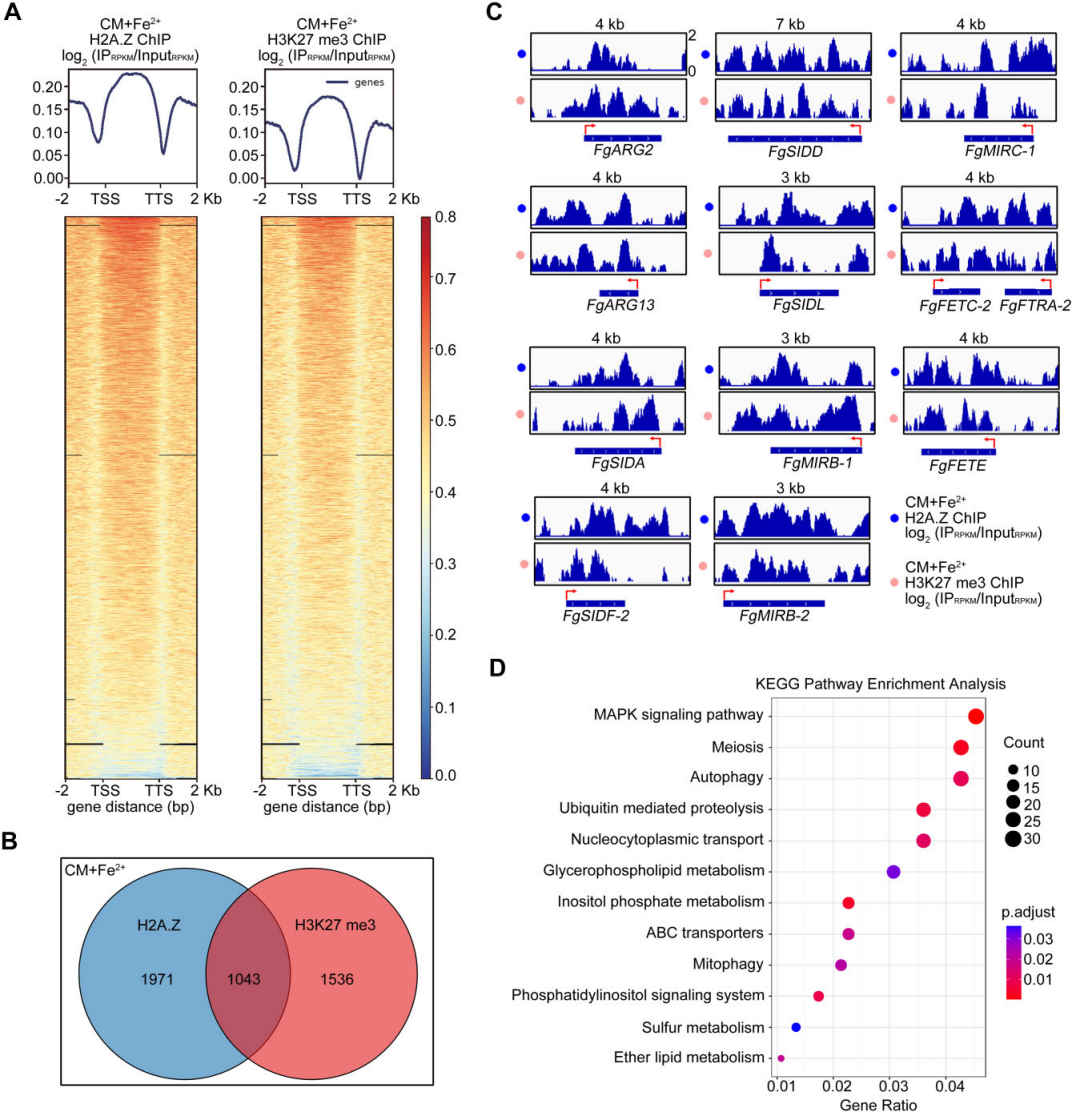
The enrichment of H2A.Z and H3K27 me3 upon iron excess. (**A**) Scatterplots and heat maps for distributions of H2A.Z and H3K27 me3 signals under iron excess treatment. TSS (transcription start site), TTS (transcription termination site). The H2A.Z and H3K27 me3 signals expand from 2 kb upstream to 2 kb downstream. (**B**) Venn diagrams showing significant overlaps of genes occupied by H2A.Z and H3K27 me3 under iron excess treatment. (**C**) ChIP-seq data were shown in the IGV (Integrative Genomics Viewer) browser. The ChIP signals were calculated as log_2_ (IP_RPKM_/Input_RPKM_) for each 10 bp. (**D**) The Kyoto Encyclopedia of Genes and Genomes (KEGG) pathway analysis of H2A.Z and H3K27 me3 overlapped target genes under iron excess treatment.

In embryonic stem cells and *Arabidopsis*, H2A.Z coordinates with H3K27 me3 to play a crucial role in transcriptional repression (81,82). Therefore, we performed ChIP-seq analyses for H2A.Z and H3K27 me3 upon iron excess. H2A.Z and H3K27 me3 showed high genome-wide co-localization (Figure 5A; Supplementary Figure S12B). A total of 3014 H2A.Z and 2579 H3K27 me3 enriched genes were identified (*p* ≤ 0.005) (Figure 5B). Intriguingly, 1043 genes were co-regulated by H2A.Z and H3K27 me3, indicating that more than 40% of H3K27 me3 enriched genes overlapped with H2A.Z (Figure 5B). Among the 1043 genes, 12 iron acquisition genes displayed significant enrichments of H2A.Z and H3K27 me3 upon iron excess (Figure 5C). In addition, A Kyoto Encyclopedia of Genes and Genomes (KEGG) analysis displayed overlapping genes involved in several important pathways, such as MAPK signaling pathway, meiosis, autophagy, ubiquitin mediated proteolysis, nucleocytoplasmic transport, glycerophospholipid metabolism, etc. (Figure 5D). These results suggest that many important biological processes are simultaneously regulated by H2A.Z and H3K27 me3 upon iron excess (Figure 5D).

### Iron-sensing of FgHapX and FgSreA is dependent on monothiol glutaredoxin FgGrx4

**Figure 6.**
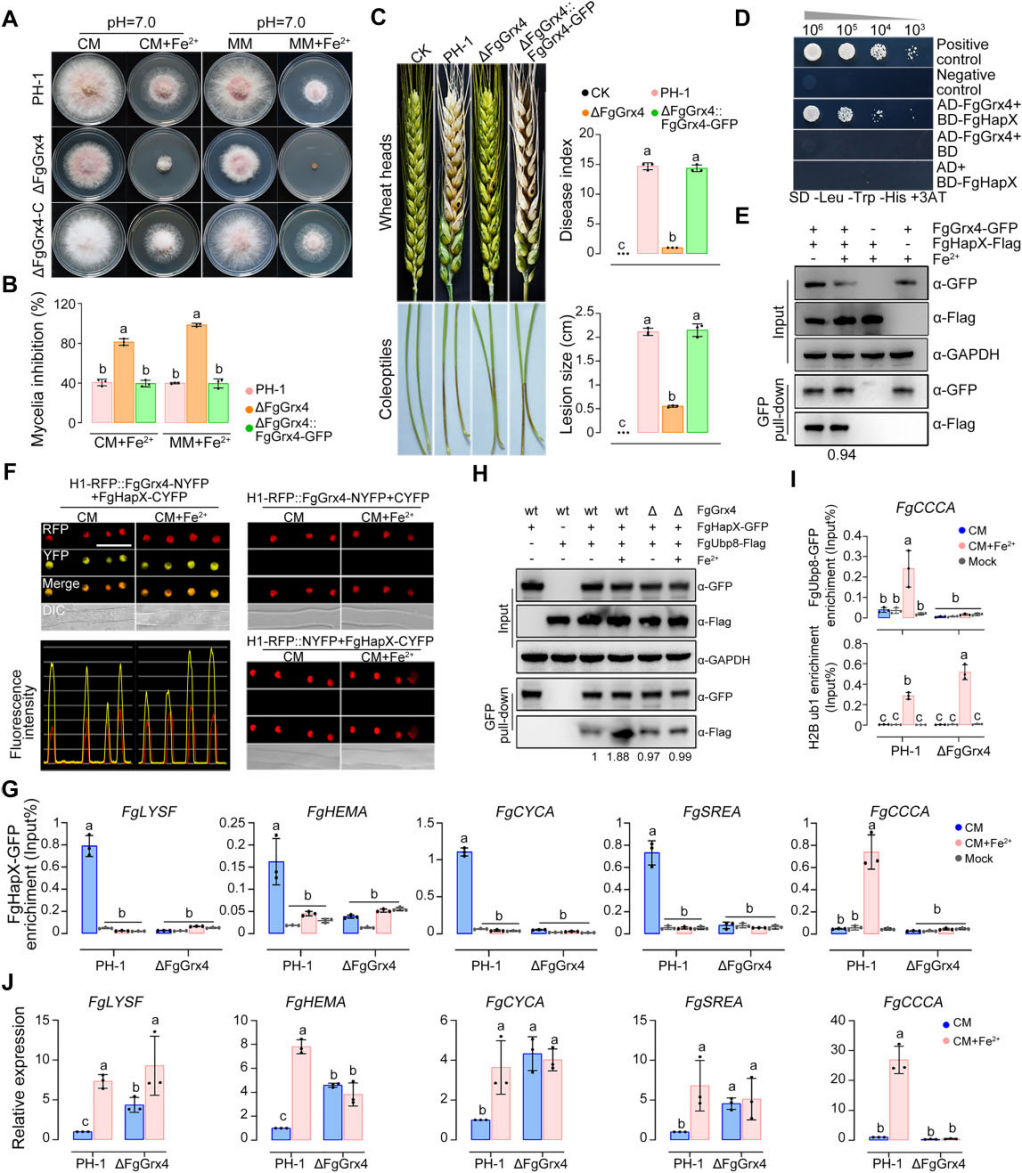
Iron-sensing of FgHapX relies on monothiol glutaredoxin FgGrx4. (A, B) Lack of FgGrx4 caused increased sensitivity to iron excess. Colony morphology was observed (**A**), and mycelial inhibition was determined (**B**) after growth on CM with or without 10 mM Fe_2_SO_4_ for 3 days. (**C**) Lack of FgGrx4 caused significantly reduced virulence on wheat heads and coleoptiles. Representative images of wheat heads were photographed, and disease index was calculated at 15 dpi. The inoculated site on each wheat head was labeled with a black dot. Representative images of coleoptiles were photographed, and lesion sizes were measured at 4 dpi. Sterile water was used as the control (CK). (**D**) FgGrx4 interacts with FgHapX in Y2H assays. Serial dilutions of yeast cells (cells/ml) transferred with the bait and prey constructs were assayed for growth on SD-Leu-Trp-His + 3AT plates. A pair of plasmids pGBKT7 (BD)-53 and pGADT7 (AD)-T was used as a positive control. A pair of plasmids pGBKT7-Lam and pGADT7-T was used as a negative control. (**E**) FgGrx4 interacts with FgHapX and the interaction was not changed by iron excess treatment in Co-IP assays. (**F**) FgGrx4 interacts with FgHapX and the interaction was not altered by iron excess treatment in bimolecular fluorescence complementation (BiFC) assays. YFP signals were observed using confocal microscopy. Bar = 10 μm. Fluorescence intensity was further evaluated by line-scan graph analysis, and the horizontal axis indicates the distance. (**G**) ChIP-qPCR assays showed that the enrichment of FgHapX-GFP at target genes promoters was dependent on FgGrx4. (**H**) The increased interaction of FgHapX and FgUbp8 upon iron excess was lost in ΔFgGrx4 in Co-IP assays. (**I**) ChIP-qPCR assays showed that deletion of FgGrx4 caused reduced enrichment of FgUbp8 and increased H2B ub1 level at the *FgCCCA* promoter. (**J**) Lack of FgGrx4 damaged the transcription of iron utilization and storage genes, and *FgSREA*. The expression level of each iron homeostasis gene in the wild type (PH-1) in CM without iron excess treatment was set to 1 and the *FgACTIN* gene was used as the internal control for normalization. In (E, H), the protein samples detected with anti-GAPDH antibody were used as a reference. The intensities of the western blotting bands were quantified with the program ImageJ. Values on the bars are the intensity of detected protein band relative to that of GAPDH band. In (G, I), the input-DNA and ChIP-DNA samples were quantified by quantitative PCR assays with corresponding primer pairs (Supplementary Table S1). ChIP signals are shown as the percentages of input and mock-DNA incubated with anti-IgG antibody as a control. In (B, C, G, I, J), mean and standard deviation were estimated with data from three independent biological replicates (marked with black or red dots, *n* = 3). Different letters indicate statistically significant differences according to the one-way ANOVA test (*P*< 0.05).

The activities of fungal iron-responsive transcription factors are regulated via iron-sulfur cluster signaling mediated by monothiol glutaredoxins (GRXs) (83). Here, we found that the FgGrx4 mutant exhibited increased sensitivity to iron excess and unaltered iron conditions (Figure 6A, B and Supplementary Figure S13). Moreover, similar to the double mutant ΔFgHapX-SreA, ΔFgGrx4 caused restricted scab symptoms only in inoculated wheat heads and coleoptiles (Figure 6C), suggesting that FgGrx4 is involved in the adaptation to iron excess during infection in *F. graminearum*.

To explore the effect of FgGrx4 on the function of FgHapX, the Y2H, Co-IP assays and BiFC assays showed that FgGrx4 interacts with FgHapX (Figure 6D–F) and that the interaction strengths were similar with or without iron excess treatment in the BiFC and Co-IP assays (Figure 6E, F). Next, the ChIP-qPCR analyses revealed that the lack of FgGrx4 completely destroyed FgHapX occupancy at the promoters of iron utilization genes and *FgSREA* without iron treatment, and impaired FgHapX enrichment at the promoter of iron storage gene *FgCCCA* with iron excess treatment (Figure 6G). Furthermore, Co-IP assays showed that the enhanced interaction between FgHapX and H2B ub1 deubiquitinase FgUbp8 upon iron excess is dependent on FgGrx4 (Figure 6H). Accordingly, the ChIP-qPCR analyses showed that the increased FgUbp8 enrichment at the *FgCCCA* promoter was compromised in ΔFgGrx4 under iron excess (Figure 6I). Meanwhile, H2B ub1 level at the *FgCCCA* promoter was elevated in ΔFgGrx4 compared to that in the wild type under iron excess (Figure 6I). In addition, qRT-PCR assays showed that iron utilization genes and *FgSREA* were upregulated in ΔFgGrx4 without iron treatment, and *FgCCCA* could not be induced in ΔFgGrx4 with iron excess treatment (Figure 6J), which is similar to the situation in ΔFgHapX (Figure 2B). These results indicate that FgGrx4 regulates the occupancy of FgHapX on target genes and its interaction with FgUbp8, subsequently controlling its transcription activity.

**Figure 7.**
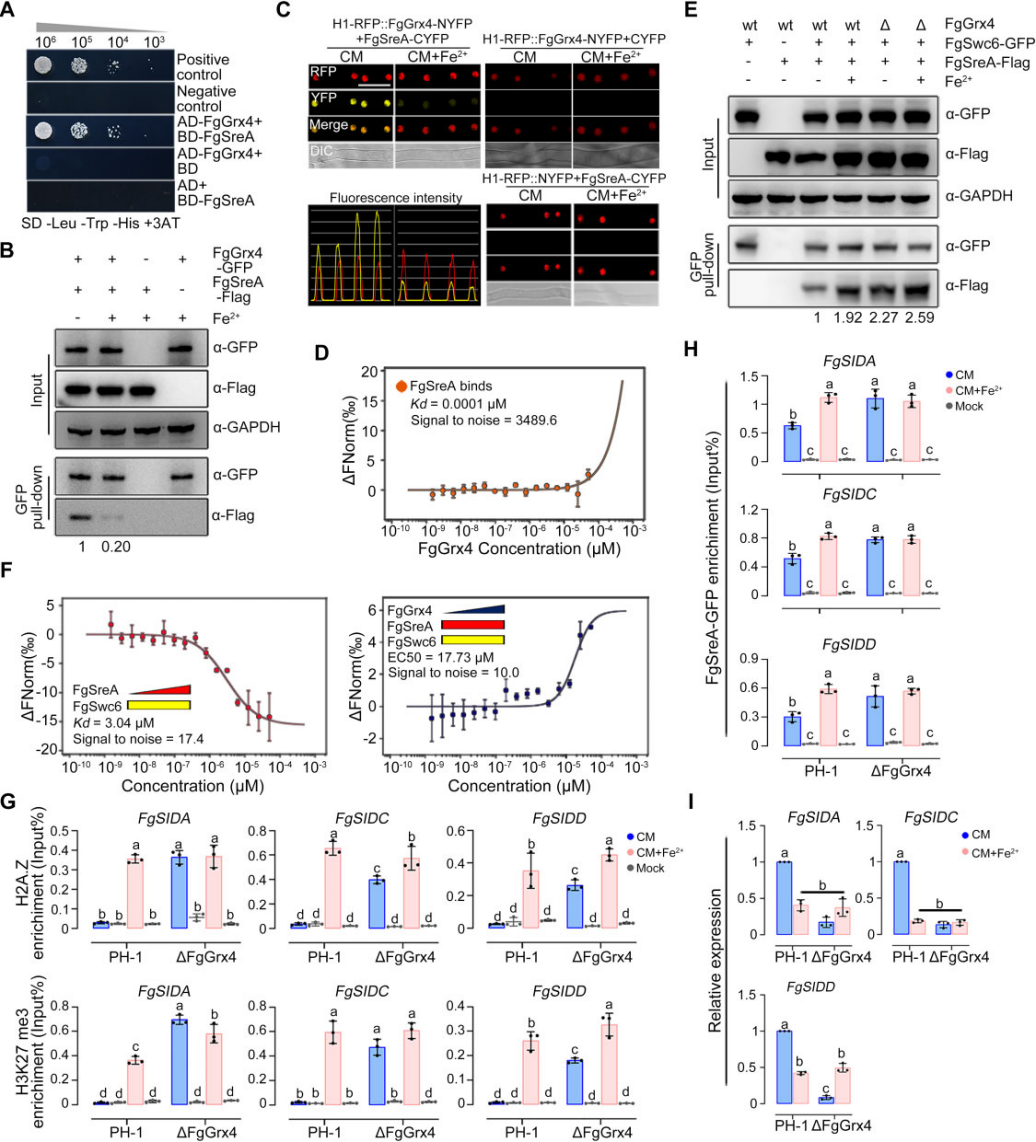
Monothiol glutaredoxin FgGrx4 transmits iron signal to FgSreA. (**A**) FgGrx4 interacts with FgSreA in Y2H assays. Serial dilutions of yeast cells (cells/ml) transferred with the bait and prey constructs were assayed for growth on SD-Leu-Trp-His + 3AT plates. A pair of plasmids pGBKT7 (BD)-53 and pGADT7 (AD)-T was used as a positive control. A pair of plasmids pGBKT7-Lam and pGADT7-T was used as a negative control. (**B**) FgGrx4 interacts with FgSreA and the interaction was dramatically decreased by iron excess treatment in Co-IP assays. (**C**) FgGrx4 interacts with FgSreA and the interaction was dramatically reduced by iron excess treatment in BiFC assays. YFP signals were observed using confocal microscopy. Bar = 10 μm. Fluorescence intensity was further evaluated by line-scan graph analysis, and the horizontal axis indicates the distance. (**D**) Microscale thermophoresis (MST) confirmed the interaction of FgSreA and FgGrx4. Labeled FgSreA protein (50 nM) was incubated with a serial concentration of FgGrx4 protein and detected by Monolith NT.115 [NT.115Pico]. An MST time of 1.5 s was used for analysis, and a *K*_d_ value was derived for this interaction. Three independent repeats were performed and data represent mean ± SE from three independent replicates (*n* = 3). (**E**) Lack of FgGrx4 enhanced the interaction of FgSreA and FgSwc6 with or without iron excess in Co-IP assays. (**F**) MST assays revealed that FgGrx4 and FgSwc6 competitively interact with FgSreA. Red dots mean FgSwc6 incubated with a serial concentration of FgSreA protein. Blue dots mean FgSwc6 incubated with the same concentration of FgSreA protein and a serial concentration of FgGrx4 protein. Two systems are detected by Monolith NT.115 [NT.115Pico]. An MST time of 1.5 s was used for analysis, and a EC50 value was derived for this competition. Three independent repeats were performed and data represent mean ± SE from three independent replicates (*n* = 3). (**G**) ChIP-qPCR assays showed that the enrichment of H2A.Z and H3K27 me3 at + 1 nucleosome of iron acquisition genes was elevated in ΔFgGrx4. (**H**) Deletion of FgGrx4 caused increased enrichment of FgSreA-GFP at the promoters of iron acquisition genes without iron treatment in ChIP-qPCR assays. (**I**) Lack of FgGrx4 caused decreased transcription of iron acquisition genes. The expression level of each gene in the wild type (PH-1) in CM without iron excess treatment was set to 1 and the *FgACTIN* gene was used as the internal control for normalization. In (B, E), the protein samples detected with anti-GAPDH antibody were used as a reference. The intensities of the western blotting bands were quantified with the program ImageJ. Values on the bars are the intensity of detected protein band relative to that of GAPDH band. In (G, H), the input-DNA and ChIP-DNA samples were quantified by quantitative PCR assays with corresponding primer pairs (Supplementary Table S1). ChIP signals are shown as the percentages of input and mock-DNA incubated with anti-IgG antibody as a control. In (G, H, I), mean and standard deviation were estimated with data from three independent biological replicates (marked with black or gray dots, *n* = 3). Different letters indicate statistically significant differences according to the one-way ANOVA test (*P*< 0.05).

To determine whether FgGrx4-mediated iron sensing also regulates the function of FgSreA upon iron excess, we detected the interaction between FgGrx4 and FgSreA. As shown in Figure 7A–D, FgGrx4 interacts with FgSreA in the Y2H, Co-IP, BiFC and MST assays, and the strength of interaction was significantly decreased upon iron excess. Given that the interaction of FgSreA and FgSwc6 was enhanced under iron excess conditions (Figure 4D), we were interested in detecting the interaction between FgSreA and FgSwc6 in ΔFgGrx4. Co-IP assays showed that deletion of FgGrx4 increased the interaction between FgSreA and FgSwc6 with or without iron excess treatment (Figure 7E). With the addition of FgGrx4, the EC50 fit curve of MST competition experiment showed an opposite direction compared with the binding experiment, suggesting that FgGrx4 was competing with FgSwc6 to bind FgSreA (Figure 7F). Accordingly, ChIP-qPCR analyses revealed that lack of FgGrx4 increased the enrichments of H2A.Z and H3K27 me3 at the +1 nucleosome of iron acquisition genes without iron treatment (Figure 7G). In addition, ChIP-qPCR analyses showed increased enrichment of FgSreA at the promoters of iron acquisition genes without iron treatment in ΔFgGrx4 (Figure 7H), which may result from the induced expression of FgSreA in ΔFgGrx4 (Figure 6J). As expected, iron acquisition genes were downregulated in ΔFgGrx4 without iron treatment (Figure 7I). Taken together, these results suggest that FgGrx4 modulates the transcription activity of FgSreA by affecting its enrichment of target genes and recruitment of the SWR1 and PRC2 complexes.

## DISCUSSION

**Figure 8.**
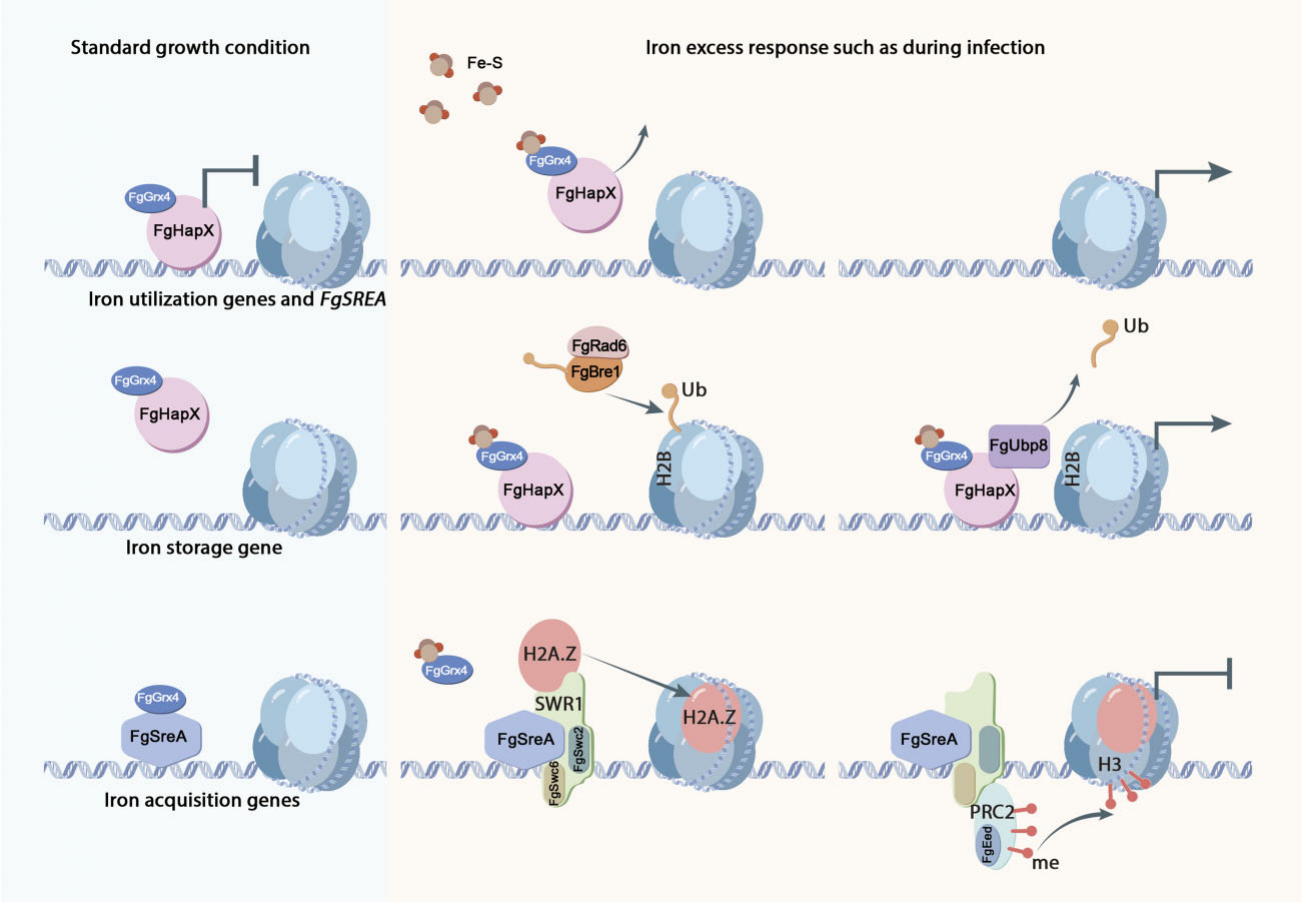
Proposed model for the molecular mechanism of FgHapX- and FgSreA-mediated iron resistance during infection in *F. graminearum*. *F. graminearum* is challenged by wheat-derived iron excess during infection. Monothiol glutaredoxin FgGrx4 senses and transmits the signal of iron excess to the transcription factor FgHapX. Under iron excess, FgHapX dissociates from the promoters of *FgSREA* and iron utilization genes, then derepresses transcription. On the other hand, FgHapX occupies the iron storage gene *FgCCCA*, facilitates H2B deub1 via interacting with ubiquitin protease FgUbp8, and consequently activates transcription. Meantime, the interaction of FgGrx4 and transcription factor FgSreA is reduced, and the dissociated FgSreA interacts with the SWR1 complex to promote H2A.Z deposition on iron acquisition genes. Subsequently, the SWR1 complex recruits the PRC2 complex to increase H3K27 me3 level on target genes, resulting in transcriptional inhibition.

In this study, FgHapX and FgSreA co-regulated adaption to wheat-derived iron excess during infection in *F. graminearum* (Figure 8). However, in the mammalian pathogens *A. fumigatus* and *C. albicans*, only HapX homologs are responsible for overcoming iron starvation during infection (8,10), whereas SreA orthologs have no role in virulence (24,84). In *C. neoformans*, Cir1 (SreA ortholog) regulates iron acquisition during infection (85) whereas HapX plays a minimal role during infection in mammalian hosts (9). In addition, the inter-regulatory model between FgHapX and FgSreA differed from that in mammalian pathogenic fungi: *FgHAPX* transcription was not changed by the deletion of FgSreA upon iron excess. In contrast, *HAPX* transcription was repressed by SreA under iron excess conditions in *A. fumigatus* (26) and *C. albicans* (10). *FgSREA* transcription is negatively regulated by FgHapX, which parallels results observed in *A. fumigatus* (26) and *C. albicans* (84). Conversely, *C. neoformans CIR1* transcription is positively regulated by HapX (9,86). These results indicate that the regulatory networks of HapX and SreA homologs during infection differ among pathogenic fungi.

HapX orthologs have been reported to activate the iron storage gene *CCCA* transcription to detoxify excessive iron in *A. fumigatus* and *V. dahlia* (23,58), but the activation mechanism is unknown. Here we demonstrate that FgHapX activates *FgCCCA* transcription by promoting H2B deub1 at the *FgCCCA* promoter (Figures 3, 8). Moreover, FgRad6- and FgBre1-regulated H2B ub1 also participate in *FgCCCA* transcriptional activation upon iron excess. In budding yeast and *Arabidopsis*, both histone H2B ub1 and deub1 are related to transcriptional activation (65–68), which is in line with our findings. In eukaryotes, H2B ub1 is highly conserved and regulates cell growth, development, cell cycle progression, virulence, response to DNA damage, cell wall and oxidative stresses (45,87). Our finding that H2B ub1/deub1 is involved in the tolerance to iron excess, broadens the functions of this histone modification in eukaryotes. In addition, H2B deubiquitination is regulated not only by the ubiquitin-specific protease Ubp8, but also by other components, Sus1, Sgf11 and Sgf73, in the DUB module in yeast (88–90). In this study, no homologs of Sus1 and Sgf11 were identified by BLAST searching the *F. graminearum* genome, reminiscent of the ascomycetous fungus *M. oryzae* (91). FgSgf73 is not involved in the regulation of H2B deubiquitination (Supplementary Figure S7), indicating that a different mode of Ubp8-regulated deubiquitination may occur in filamentous ascomycetous fungi. Taken together, we report for the first time the HapX-mediated transcriptional activation mechanism of iron storage genes at the chromatin level in fungi.

In this study, the CBC is required for the functions of FgHapX in iron homeostasis, which is similar to the reports in *A. fumigates* (20,23). Phenotypic analyses showed that *FgHapB^KD^* displayed difference in defects of mycelial growth, virulence, mycotoxin DON production, and asexual development compared with ΔFgHapX (Supplementary Figure S14), indicating that the CBC has FgHapX-independent functions in *F. graminearum*. Interestingly, the CBC is essential in *F. graminearum*, which contrasts other fungal species such as *A. fumigates, A. nidulans* and *Candida glabrata* (9,92–96). Moreover, FgHapX interacts with both FgHapB and FgHapC, but not with FgHapE (Supplementary Figure S5F, G), in contrast, the N-terminal part of HapE is required for interaction with HapX in *A. nidulans* (97). These results indicate that the functions of CBC and the interaction mode of CBC with HapX might vary among fungi.

In fungi, the function of SreA in inhibiting iron acquisition gene expression is conserved (24,59,98,99). Our study is the first to reveal the transcriptional repression mechanism of iron acquisition genes regulated by SreA in an important pathogenic fungus. FgSreA facilitates the enrichments of H2A.Z and H3K27 me3 at the + 1 nucleosomes of iron acquisition genes to decrease nucleosome accessibility, subsequently repressing their transcription upon iron excess (Figure 4, 8). To date, studies on the histone variant H2A.Z have been performed in several mammals and plants and fungi (72,76,100,101). The deposition and eviction of H2A.Z is involved in a diverse range of biological processes, including growth (70,102), genome stability (103), DNA repair (104–106), response to temperature fluctuations (107,108), drought and oxidative stress (109), pathogen infection, and nutrient stress via transcriptional regulation (69). In this study, we found that the H2A.Z regulates iron homeostasis in *F. graminearum*, which is important for the functions of H2A.Z in eukaryotic organisms.

H2A.Z usually regulates gene expression through collaborating with other histone marks, particularly those on H3. Here, we found that the H2A.Z repressed the transcription via regulating the biogenesis of H3K27 me3 at iron acquisition genes. Moreover, ChIP-seq analyses of H2A.Z and H3K27 me3 revealed that besides iron acquisition genes, genes involved in several important pathways were simultaneously regulated by H2A.Z and H3K27 me3 (Figure 5D). Similar findings have been reported in other eukaryotic organisms. In embryonic stem cells, H2A.Z was reported to localize exclusively at H3K27 me3-target genes and mediate the targeting of H3K27 me3 and repression of these genes (81,110). In *Arabidopsis*, H2A.Z is preferentially associated with H3K27 me3 at enhancers, and represses enhancer activity by promoting H3K27 me3, resulting in gene repression (82). However, in *Neurospora crassa*, H2A.Z and H3K27 me3 do not co-localize in the genome, and deletion of *N. crassa* H2A.Z caused reduced expression of *EED*, then resulting in decreased level of PRC2-mediated H3K27 me3 (101). Differently, FgH2A.Z was not enriched on *FgEED* and transcription of *FgEED* was not altered by lack of either of the SWR1 complex components FgSwc2 or FgSwc6 (Supplementary Figure S15). Notably, our study found that H2A.Z deposition modulated the presence of H3K27 me3 via the interaction of SWR1 complex and FgEed, which will widen understanding of the correlation between H2A.Z and H3K27 me3. In addition, H2A.Z deposition has been found to be linked to both transcriptional activation and repression; moreover, promotion or repression of transcription is dependent on its localization within the gene (69,82,103,111,112). Some studies have reported that H2A.Z located at the first nucleosome after the transcription start site is associated with transcriptional activation, while its presence in nucleosomes within gene bodies is correlated with transcriptional repression (69,111). In the current study, we found that H2A.Z is located at the + 1 nucleosome of iron acquisition genes but inhibits their transcription upon iron excess. Similar findings have been reported in *Arabidopsis*, *Caenorhabditis elegans* and *Drosophila* (71,82,113,114), supporting a positive correlation between H2A.Z location at the +1 nucleosome and transcriptional repression in eukaryotic organisms.

Grx3/4-type monothiol glutaredoxins play important roles in controlling iron homeostasis because of their conserved roles in [2Fe-2S] cluster sensing and trafficking in pathogenic fungi (34,83,115,116). As reported for *A. fumigatus* and *C. albicans* (117,118), FgGrx4 interacts with both FgHapX and FgSreA. However, the interaction of HapX/SreA and Grx3/4 had different effects on the functions of these two transcription factors in fungi. In *A. fumigatus*, although HapX interacts with GrxD (Grx3/4 ortholog), sensing iron excess by HapX is independent of GrxD (33); whereas in *C. albicans*, the interaction of Grx3 and Hap43 (HapX ortholog) affects nucleocytoplasmic shuttling of Hap43 in response to iron (118). In our study, the interaction of FgGrx4 and FgHapX controlled the DNA binding ability and deubiquitinase FgUbp8 recruitment of FgHapX upon iron excess. Regarding the interaction of SreA and Grx3/4, *C. albicans* Grx3/4 regulates the iron-responsive enrichment of Sfu1 (SreA ortholog) on the extracellular haem receptor-encoding gene *RBT5* (118). However, we found that lack of FgGrx4 did not affect FgSreA iron-responsive enrichment on iron acquisition genes; in contrast, it dramatically increased FgSreA occupancy without iron treatment, suggesting constitutive repression of iron acquisition genes by FgSreA as reported for *A. fumigatus* (117,118). Interestingly, the interaction of FgGrx4 with FgSreA dramatically decreased under iron excess, and the dissociated FgSreA subsequently interacted with the SWR1 complex. Accordingly, deletion of FgGrx4 increased the interaction between FgSreA and the SWR1 complex. To our knowledge, this is the first study to report that monothiol glutaredoxin regulates the transcriptional activity of HapX and SreA by affecting the recruitment of chromatin structure modulators in fungi.

In conclusion, we uncovered the molecular mechanism of iron excess adaptation mediated by the transcription factors FgHapX and FgSreA, which is crucial during *F. graminearum* infection (Figure 8). Wheat produces excessive iron to prevent *F. graminearum* infection. FgGrx4 senses and transmits the signal of iron to FgHapX and FgSreA. Furthermore, FgHapX derepresses *FgSREA* and iron utilization genes via dissociation from their promoters and activates the iron storage gene by promoting H2B deub1. Meanwhile, FgSreA inhibits iron acquisition genes by facilitating the deposition of H2A.Z and H3K27 me3. Importantly, this is the first report to address that the histone modifications H2B deub1 and H3K27 me3, as well as the deposition of histone variant H2A.Z, co-regulate iron homeostasis in eukaryotic organisms.

## Supplementary Material

gkad708_Supplemental_Files

## Data Availability

Relevant data supporting the findings of this study are available in this article and its Supplementary Information files. The ChIP-seq and RNA-seq raw and processed data have been deposited in the National Center for Biotechnology Information BioProject under the accession number PRJNA786942. The mass spectrometry proteomics data have been deposited to the ProteomeXchange Consortium (http://proteomecentral.proteomexchange.org) with the dataset identifier PXD035434. Source data are provided with this paper.

## References

[1] Pierre J.L. , Fontecave M., Crichton R.R. Chemistry for an essential biological process: the reduction of ferric iron. Biometals. 2002; 15:341–346. 12405527 10.1023/a:1020259021641

[2] Kaplan C.D. , Kaplan J. Iron acquisition and transcriptional regulation. Chem. Rev. 2009; 109:4536–4552. 19705827 10.1021/cr9001676

[3] Ding C. , Festa R.A., Sun T.S., Wang Z.Y. Iron and copper as virulence modulators in human fungal pathogens. Mol. Microbiol. 2014; 93:10–23. 24851950 10.1111/mmi.12653

[4] Cassat J.E. , Skaar E.P. Iron in infection and immunity. Cell Host Microbe. 2013; 13:509–519. 23684303 10.1016/j.chom.2013.04.010 PMC3676888

[5] Ganz T. , Nemeth E. Iron homeostasis in host defence and inflammation. Nat. Rev. Immunol. 2015; 15:500–510. 26160612 10.1038/nri3863 PMC4801113

[6] Verbon E.H. , Trapet P.L., Stringlis I.A., Kruijs S., Bakker P., Pieterse C.M.J. Iron and Immunity. Annu. Rev. Phytopathol. 2017; 55:355–375. 28598721 10.1146/annurev-phyto-080516-035537

[7] Kieu N.P. , Aznar A., Segond D., Rigault M., Simond-Côte E., Kunz C., Soulie M.C., Expert D., Dellagi A. Iron deficiency affects plant defence responses and confers resistance to *Dickeya dadantii* and *Botrytis cinerea*. Mol. Plant Pathol. 2012; 13:816–827. 22375884 10.1111/j.1364-3703.2012.00790.x PMC6638873

[8] Schrettl M. , Beckmann N., Varga J., Heinekamp T., Jacobsen I.D., Jöchl C., Moussa T.A., Wang S., Gsaller F., Blatzer M. et al. HapX-mediated adaption to iron starvation is crucial for virulence of *Aspergillus fumigatus*. PLoS Pathog. 2010; 6:e1001124. 20941352 10.1371/journal.ppat.1001124 PMC2947994

[9] Jung W.H. , Saikia S., Hu G., Wang J., Fung C.K., D'Souza C., White R., Kronstad J.W HapX positively and negatively regulates the transcriptional response to iron deprivation in *Cryptococcus neoformans*. PLoS Pathog. 2010; 6:e1001209. 21124817 10.1371/journal.ppat.1001209 PMC2991262

[10] Hsu P.C. , Yang C.Y., Lan C.Y. *Candida albicans* Hap43 is a repressor induced under low-iron conditions and is essential for iron-responsive transcriptional regulation and virulence. Euk. Cell. 2011; 10:207–225. 10.1128/EC.00158-10 PMC3067405 21131439

[11] Franza T. , Expert D Role of iron homeostasis in the virulence of phytopathogenic bacteria: an ‘à la carte’ menu. Mol. Plant Pathol. 2013; 14:429–438. 23171271 10.1111/mpp.12007 PMC6638640

[12] Eichhorn H. , Lessing F., Winterberg B., Schirawski J., Kämper J., Müller P., Kahmann R. A ferroxidation/permeation iron uptake system is required for virulence in *Ustilago maydis*. Plant Cell. 2006; 18:3332–3345. 17138696 10.1105/tpc.106.043588 PMC1693961

[13] López-Berges M.S. , Capilla J., Turrà D., Schafferer L., Matthijs S., Jöchl C., Cornelis P., Guarro J., Haas H., Di Pietro A. HapX-mediated iron homeostasis is essential for rhizosphere competence and virulence of the soilborne pathogen *Fusarium oxysporum*. Plant Cell. 2012; 24:3805–3822. 22968717 10.1105/tpc.112.098624 PMC3480304

[14] Liu G. , Greenshields D.L., Sammynaiken R., Hirji R.N., Selvaraj G., Wei Y. Targeted alterations in iron homeostasis underlie plant defense responses. J. Cell Sci. 2007; 120:596–605. 17244651 10.1242/jcs.001362

[15] Ye F. , Albarouki E., Lingam B., Deising H.B., von Wirén N. An adequate Fe nutritional status of maize suppresses infection and biotrophic growth of *Colletotrichum graminicola*. Physiol. Plant. 2014; 151:280–292. 24512386 10.1111/ppl.12166

[16] Franken A.C. , Lechner B.E., Werner E.R., Haas H., Lokman B.C., Ram A.F., van den Hondel C.A., de Weert S., Punt P.J. Genome mining and functional genomics for siderophore production in *Aspergillus niger*. Brief. Funct. Genomics. 2014; 13:482–492. 25062661 10.1093/bfgp/elu026

[17] Haas H. Fungal siderophore metabolism with a focus on *Aspergillus fumigatus*. Nat. Prod. Rep. 2014; 31:1266–1276. 25140791 10.1039/c4np00071d PMC4162504

[18] Wang J. , Pantopoulos K. Regulation of cellular iron metabolism. Biochem. J. 2011; 434:365–381. 21348856 10.1042/BJ20101825 PMC3048577

[19] Oberegger H. , Schoeser M., Zadra I., Abt B., Haas H. SREA is involved in regulation of siderophore biosynthesis, utilization and uptake in *Aspergillus nidulans*. Mol. Microbiol. 2001; 41:1077–1089. 11555288 10.1046/j.1365-2958.2001.02586.x

[20] Hortschansky P. , Eisendle M., Al-Abdallah Q., Schmidt A.D., Bergmann S., Thön M., Kniemeyer O., Abt B., Seeber B., Werner E.R. et al. Interaction of HapX with the CCAAT-binding complex–a novel mechanism of gene regulation by iron. EMBO J. 2007; 26:3157–3168. 17568774 10.1038/sj.emboj.7601752 PMC1914100

[21] Kröber A. , Scherlach K., Hortschansky P., Shelest E., Staib P., Kniemeyer O., Brakhage A.A. HapX mediates iron homeostasis in the pathogenic dermatophyte *arthroderma benhamiae* but is dispensable for virulence. PLoS One. 2016; 11:e0150701. 26960149 10.1371/journal.pone.0150701 PMC4784894

[22] Wang Z. , Ma T., Huang Y., Wang J., Chen Y., Kistler H.C., Ma Z., Yin Y. A fungal ABC transporter FgAtm1 regulates iron homeostasis via the transcription factor cascade FgAreA-HapX. PLoS Pathog. 2019; 15:e1007791. 31545842 10.1371/journal.ppat.1007791 PMC6788720

[23] Gsaller F. , Hortschansky P., Beattie S.R., Klammer V., Tuppatsch K., Lechner B.E., Rietzschel N., Werner E.R., Vogan A.A., Chung D. et al. The Janus transcription factor HapX controls fungal adaptation to both iron starvation and iron excess. EMBO J. 2014; 33:2261–2276. 25092765 10.15252/embj.201489468 PMC4232046

[24] Schrettl M. , Kim H.S., Eisendle M., Kragl C., Nierman W.C., Heinekamp T., Werner E.R., Jacobsen I., Illmer P., Yi H. et al. SreA-mediated iron regulation in *Aspergillus fumigatus*. Mol. Microbiol. 2008; 70:27–43. 18721228 10.1111/j.1365-2958.2008.06376.x PMC2610380

[25] Gsaller F. , Eisendle M., Lechner B.E., Schrettl M., Lindner H., Müller D., Geley S., Haas H. The interplay between vacuolar and siderophore-mediated iron storage in *Aspergillus fumigatus*. Metallomics. 2012; 4:1262–1270. 23151814 10.1039/c2mt20179h

[26] Schrettl M. , Haas H. Iron homeostasis–Achilles' heel of Aspergillus fumigatus?. Curr. Opin. Microbiol. 2011; 14:400–405. 21724450 10.1016/j.mib.2011.06.002 PMC3162135

[27] Furukawa T. , Scheven M.T., Misslinger M., Zhao C., Hoefgen S., Gsaller F., Lau J., Jöchl C., Donaldson I., Valiante V. et al. The fungal CCAAT-binding complex and HapX display highly variable but evolutionary conserved synergetic promoter-specific DNA recognition. Nucleic Acids Res. 2020; 48:3567–3590. 32086516 10.1093/nar/gkaa109 PMC7144946

[28] Li B. , Carey M., Workman J.L. The role of chromatin during transcription. Cell. 2007; 128:707–719. 17320508 10.1016/j.cell.2007.01.015

[29] Dubey A. , Jeon J. Epigenetic regulation of development and pathogenesis in fungal plant pathogens. Mol. Plant Pathol. 2017; 18:887–898. 27749982 10.1111/mpp.12499 PMC6638268

[30] Sood V. , Cajigas I., D’Urso A., Light W.H., Brickner J.H. Epigenetic transcriptional memory of GAL genes depends on growth in glucose and the Tup1 transcription factor in *saccharomyces cerevisiae*. Genetics. 2017; 206:1895–1907. 28607146 10.1534/genetics.117.201632 PMC5560796

[31] Mai H.J. , Pateyron S., Bauer P. Iron homeostasis in *Arabidopsis thaliana*: transcriptomic analyses reveal novel FIT-regulated genes, iron deficiency marker genes and functional gene networks. BMC Plant Biol. 2016; 16:211. 27716045 10.1186/s12870-016-0899-9 PMC5048462

[32] Gao F. , Robe K., Gaymard F., Izquierdo E., Dubos C. The transcriptional control of iron homeostasis in plants: a tale of bHLH transcription factors?. Front. Plant Sci. 2019; 10:6. 30713541 10.3389/fpls.2019.00006 PMC6345679

[33] Misslinger M. , Hortschansky P., Brakhage A.A., Haas H. Fungal iron homeostasis with a focus on *Aspergillus fumigatus*. Biochim. Biophys. Acta. Mol. Cell Res. 2021; 1868:118885. 33045305 10.1016/j.bbamcr.2020.118885

[34] Talib E.A. , Outten C.E. Iron-sulfur cluster biogenesis, trafficking, and signaling: roles for CGFS glutaredoxins and BolA proteins. Biochim. Biophys. Acta. Mol. Cell Res. 2021; 1868:118847. 32910989 10.1016/j.bbamcr.2020.118847 PMC7837452

[35] Park E.Y. , Tsuyuki K.M., Hu F., Lee J., Jeong J. PRC2-Mediated H3K27me3 contributes to transcriptional regulation of FIT-dependent iron deficiency response. Front. Plant Sci. 2019; 10:627. 31156682 10.3389/fpls.2019.00627 PMC6532572

[36] Srivastav M.K. , Agarwal N., Poonia P., Natarajan K. Interplay between transcriptional regulators and the SAGA chromatin modifying complex fine-tune iron homeostasis. J. Biol. Chem. 2021; 297:100727. 33933457 10.1016/j.jbc.2021.100727 PMC8217685

[37] Gu Q. , Wang Y., Zhao X., Yuan B., Zhang M., Tan Z., Zhang X., Chen Y., Wu H., Luo Y. et al. Inhibition of histone acetyltransferase GCN5 by a transcription factor FgPacC controls fungal adaption to host-derived iron stress. Nucleic Acids Res. 2022; 50:6190–6210. 35687128 10.1093/nar/gkac498 PMC9226496

[38] Dean R. , Van Kan J.A., Pretorius Z.A., Hammond-Kosack K.E., Di Pietro A., Spanu P.D., Rudd J.J., Dickman M., Kahmann R., Ellis J. et al. The top 10 fungal pathogens in molecular plant pathology. Mol. Plant Pathol. 2012; 13:414–430. 22471698 10.1111/j.1364-3703.2011.00783.x PMC6638784

[39] Bennett J.W. , Klich M Mycotoxins. Clin. Microbiol. Rev. 2003; 16:497–516. 12857779 10.1128/CMR.16.3.497-516.2003 PMC164220

[40] Jian Y. , Liu Z., Wang H., Chen Y., Yin Y., Zhao Y., Ma Z. Interplay of two transcription factors for recruitment of the chromatin remodeling complex modulates fungal nitrosative stress response. Nat. Commun. 2021; 12:2576. 33958593 10.1038/s41467-021-22831-8 PMC8102577

[41] Liu Z. , Jian Y., Chen Y., Kistler H.C., He P., Ma Z., Yin Y. A phosphorylated transcription factor regulates sterol biosynthesis in *Fusarium graminearum*. Nat. Commun. 2019; 10:1228. 30874562 10.1038/s41467-019-09145-6 PMC6420630

[42] Kamihara Y. , Takada K., Sato T., Kawano Y., Murase K., Arihara Y., Kikuchi S., Hayasaka N., Usami M., Iyama S. et al. The iron chelator deferasirox induces apoptosis by targeting oncogenic Pyk2/β-catenin signaling in human multiple myeloma. Oncotarget. 2016; 7:64330–64341. 27602957 10.18632/oncotarget.11830 PMC5325446

[43] Chaudhary K. , Promsote W., Ananth S., Veeranan-Karmegam R., Tawfik A., Arjunan P., Martin P., Smith S.B., Thangaraju M., Kisselev O. et al. Iron overload accelerates the progression of diabetic retinopathy in association with increased retinal renin expression. Sci. Rep. 2018; 8:3025. 29445185 10.1038/s41598-018-21276-2 PMC5813018

[44] Riemer J. , Hoepken H.H., Czerwinska H., Robinson S.R., Dringen R. Colorimetric ferrozine-based assay for the quantitation of iron in cultured cells. Anal. Biochem. 2004; 331:370–375. 15265744 10.1016/j.ab.2004.03.049

[45] Ma T. , Zhang L., Wang M., Li Y., Jian Y., Wu L., Kistler H.C., Ma Z., Yin Y. Plant defense compound triggers mycotoxin synthesis by regulating H2B ub1 and H3K4 me2/3 deposition. New Phytol. 2021; 232:2106–2123. 34480757 10.1111/nph.17718 PMC9293436

[46] Gardiner D.M. , Osborne S., Kazan K., Manners J.M. Low pH regulates the production of deoxynivalenol by *fusarium graminearum*. Microbiology (Reading). 2009; 155:3149–3156. 19497949 10.1099/mic.0.029546-0

[47] Yun Y. , Liu Z., Zhang J., Shim W.B., Chen Y., Ma Z. The MAPKK FgMkk1 of *fusarium graminearum* regulates vegetative differentiation, multiple stress response, and virulence via the cell wall integrity and high-osmolarity glycerol signaling pathways. Environ. Microbiol. 2014; 16:2023–2037. 24237706 10.1111/1462-2920.12334

[48] Lee J. , Son H., Lee S., Park A.R., Lee Y.W. Development of a conditional gene expression system using a zearalenone-inducible promoter for the ascomycete fungus Gibberella zeae. Appl. Environ. Microbiol. 2010; 76:3089–3096. 20348311 10.1128/AEM.02999-09 PMC2869113

[49] Long Q. , Du M., Long J., Xie Y., Zhang J., Xu L., He Y., Li Q., Chen S., Zou X. Transcription factor WRKY22 regulates canker susceptibility in sweet orange (Citrus sinensis Osbeck) by enhancing cell enlargement and CsLOB1 expression. Hortic Res. 2021; 8:50. 33642585 10.1038/s41438-021-00486-2 PMC7917094

[50] Luo Y.X. , Hou X.M., Zhang C.J., Tan L.M., Shao C.R., Lin R.N., Su Y.N., Cai X.W., Li L., Chen S. et al. A plant-specific SWR1 chromatin-remodeling complex couples histone H2A.Z deposition with nucleosome sliding. EMBO J. 2020; 39:e102008. 32115743 10.15252/embj.2019102008 PMC7110101

[51] Kaufmann K. , Muiño J.M., Østerås M., Farinelli L., Krajewski P., Angenent G.C. Chromatin immunoprecipitation (ChIP) of plant transcription factors followed by sequencing (ChIP-SEQ) or hybridization to whole genome arrays (ChIP-CHIP). Nat. Protoc. 2010; 5:457–472. 20203663 10.1038/nprot.2009.244

[52] Chen S. , Zhou Y., Chen Y., Gu J. fastp: an ultra-fast all-in-one FASTQ preprocessor. Bioinformatics. 2018; 34:i884–i890. 30423086 10.1093/bioinformatics/bty560 PMC6129281

[53] Langmead B. , Salzberg S.L. Fast gapped-read alignment with Bowtie 2. Nat. Methods. 2012; 9:357–359. 22388286 10.1038/nmeth.1923 PMC3322381

[54] Tarasov A. , Vilella A.J., Cuppen E., Nijman I.J., Prins P. Sambamba: fast processing of NGS alignment formats. Bioinformatics. 2015; 31:2032–2034. 25697820 10.1093/bioinformatics/btv098 PMC4765878

[55] Ramírez F. , Ryan D.P., Grüning B., Bhardwaj V., Kilpert F., Richter A.S., Heyne S., Dündar F., Manke T. deepTools2: a next generation web server for deep-sequencing data analysis. Nucleic Acids Res. 2016; 44:W160–W165. 27079975 10.1093/nar/gkw257 PMC4987876

[56] Kaster M. , Laubinger S. Determining nucleosome position at individual loci after biotic stress using MNase-qPCR. Methods Mol. Biol. 2016; 1398:357–372. 26867638 10.1007/978-1-4939-3356-3_29

[57] Lemanceau P. , Expert D., Gaymard F., Bakker P., Briat J.F. Role of iron in plant-microbe interaction. Adv. Bot. Res. 2010; 51:491–549.

[58] Wang Y. , Deng C., Tian L., Xiong D., Tian C., Klosterman S.J. The transcription factor VdHapX controls iron homeostasis and is crucial for virulence in the vascular pathogen *Verticillium Dahliae*. mSphere. 2018; 3:e00400-18. 30185514 10.1128/mSphere.00400-18 PMC6126142

[59] Chung K.R. , Wu P.C., Chen Y.K., Yago J.I. The siderophore repressor SreA maintains growth, hydrogen peroxide resistance, and cell wall integrity in the phytopathogenic fungus *Alternaria alternata*. Fung. Genet. Biol. 2020; 139:103384. 10.1016/j.fgb.2020.103384 32278718

[60] Ehrensberger K.M. , Bird A.J. Hammering out details: regulating metal levels in eukaryotes. Trends Biochem. Sci. 2011; 36:524–531. 21840721 10.1016/j.tibs.2011.07.002

[61] Haas H. , Eisendle M., Turgeon B.G. Siderophores in fungal physiology and virulence. Annu. Rev. Phytopathol. 2008; 46:149–187. 18680426 10.1146/annurev.phyto.45.062806.094338

[62] Köhler A. , Pascual-García P., Llopis A., Zapater M., Posas F., Hurt E., Rodríguez-Navarro S. The mRNA export factor Sus1 is involved in spt/Ada/Gcn5 acetyltransferase-mediated H2B deubiquitinylation through its interaction with Ubp8 and Sgf11. Mol. Biol. Cell. 2006; 17:4228–4236. 16855026 10.1091/mbc.E06-02-0098 PMC1635344

[63] Zhang X.Y. , Varthi M., Sykes S.M., Phillips C., Warzecha C., Zhu W., Wyce A., Thorne A.W., Berger S.L., McMahon S.B. The putative cancer stem cell marker USP22 is a subunit of the human SAGA complex required for activated transcription and cell-cycle progression. Mol. Cell. 2008; 29:102–111. 18206973 10.1016/j.molcel.2007.12.015 PMC2254522

[64] Spedale G. , Timmers H.T., Pijnappel W.W. ATAC-king the complexity of SAGA during evolution. Genes Dev. 2012; 26:527–541. 22426530 10.1101/gad.184705.111 PMC3315114

[65] Henry K.W. , Wyce A., Lo W.S., Duggan L.J., Emre N.C., Kao C.F., Pillus L., Shilatifard A., Osley M.A., Berger S.L. Transcriptional activation via sequential histone H2B ubiquitylation and deubiquitylation, mediated by SAGA-associated Ubp8. Genes Dev. 2003; 17:2648–2663. 14563679 10.1101/gad.1144003 PMC280615

[66] Wyce A. , Xiao T., Whelan K.A., Kosman C., Walter W., Eick D., Hughes T.R., Krogan N.J., Strahl B.D., Berger S.L. H2B ubiquitylation acts as a barrier to Ctk1 nucleosomal recruitment prior to removal by Ubp8 within a SAGA-related complex. Mol. Cell. 2007; 27:275–288. 17643376 10.1016/j.molcel.2007.01.035

[67] Gu X. , Jiang D., Wang Y., Bachmair A., He Y. Repression of the floral transition via histone H2B monoubiquitination. Plant J. 2009; 57:522–533. 18980658 10.1111/j.1365-313X.2008.03709.x

[68] Schmitz R.J. , Tamada Y., Doyle M.R., Zhang X., Amasino R.M. Histone H2B deubiquitination is required for transcriptional activation of FLOWERING LOCUS C and for proper control of flowering in *Arabidopsis*. Plant Physiol. 2009; 149:1196–1204. 19091875 10.1104/pp.108.131508 PMC2633843

[69] Sura W. , Kabza M., Karlowski W.M., Bieluszewski T., Kus-Slowinska M., Pawełoszek Ł., Sadowski J., Ziolkowski P.A. Dual role of the histone variant H2A.Z in transcriptional regulation of stress-response genes. Plant Cell. 2017; 29:791–807. 28258158 10.1105/tpc.16.00573 PMC5435421

[70] Chen Z. , Zehraoui E., Atanasoff-Kardjalieff A.K., Strauss J., Studt L., Ponts N. Effect of H2A.Z deletion is rescued by compensatory mutations in *Fusarium graminearum*. PLoS Genet. 2020; 16:e1009125. 33091009 10.1371/journal.pgen.1009125 PMC7608984

[71] Mavrich T.N. , Jiang C., Ioshikhes I.P., Li X., Venters B.J., Zanton S.J., Tomsho L.P., Qi J., Glaser R.L., Schuster S.C. et al. Nucleosome organization in the *Drosophila* genome. Nature. 2008; 453:358–362. 18408708 10.1038/nature06929 PMC2735122

[72] Barski A. , Cuddapah S., Cui K., Roh T.Y., Schones D.E., Wang Z., Wei G., Chepelev I., Zhao K. High-resolution profiling of histone methylations in the human genome. Cell. 2007; 129:823–837. 17512414 10.1016/j.cell.2007.05.009

[73] Lantermann A.B. , Straub T., Strålfors A., Yuan G.C., Ekwall K., Korber P. Schizosaccharomyces pombe genome-wide nucleosome mapping reveals positioning mechanisms distinct from those of *Saccharomyces cerevisiae*. Nat. Struct. Mol. Biol. 2010; 17:251–257. 20118936 10.1038/nsmb.1741

[74] Wu W.H. , Alami S., Luk E., Wu C.H., Sen S., Mizuguchi G., Wei D., Wu C. Swc2 is a widely conserved H2AZ-binding module essential for ATP-dependent histone exchange. Nat. Struct. Mol. Biol. 2005; 12:1064–1071. 16299513 10.1038/nsmb1023

[75] Willhoft O. , GhoneimM., LinC.L., ChuaE.Y.D., WilkinsonM., ChabanY., AyalaR., McCormackE.A., OclooL., RuedaD.S.et al. Structure and dynamics of the yeast SWR1-nucleosome complex. Science. 2018; 362:eaat7716.3030991810.1126/science.aat7716

[76] Gómez-Zambrano Á. , Merini W., Calonje M. The repressive role of *Arabidopsis* H2A.Z in transcriptional regulation depends on AtBMI1 activity. Nat. Commun. 2019; 10:2828. 31249301 10.1038/s41467-019-10773-1 PMC6597585

[77] Schuettengruber B. , Bourbon H.M., Di Croce L., Cavalli G. Genome regulation by polycomb and trithorax: 70 years and counting. Cell. 2017; 171:34–57. 28938122 10.1016/j.cell.2017.08.002

[78] Chen S. , Jiao L., Liu X., Yang X., Liu X. A dimeric structural scaffold for PRC2-PCL targeting to CpG island chromatin. Mol. Cell. 1267; 77:1265–1278. 10.1016/j.molcel.2019.12.019 PMC7571800 31959557

[79] Tang G. , Yuan J., Wang J., Zhang Y.Z., Xie S.S., Wang H., Tao Z., Liu H., Kistler H.C., Zhao Y. et al. *Fusarium* BP1 is a reader of H3K27 methylation. Nucleic Acids Res. 2021; 49:10448–10464. 34570240 10.1093/nar/gkab844 PMC8501951

[80] Connolly L.R. , Smith K.M., Freitag M. The fusarium graminearum histone H3 K27 methyltransferase KMT6 regulates development and expression of secondary metabolite gene clusters. PLoS Genet. 2013; 9:e1003916. 24204317 10.1371/journal.pgen.1003916 PMC3814326

[81] Hu G. , Cui K., Northrup D., Liu C., Wang C., Tang Q., Ge K., Levens D., Crane-Robinson C., Zhao K. H2A.Z facilitates access of active and repressive complexes to chromatin in embryonic stem cell self-renewal and differentiation. Cell Stem Cell. 2013; 12:180–192. 23260488 10.1016/j.stem.2012.11.003 PMC3570599

[82] Dai X. , Bai Y., Zhao L., Dou X., Liu Y., Wang L., Li Y., Li W., Hui Y., Huang X. et al. H2A.Z represses gene expression by modulating promoter nucleosome structure and enhancer histone modifications in *arabidopsis*. Mol. Plant. 2017; 10:1274–1292. 28951178 10.1016/j.molp.2017.09.007

[83] Gupta M. , Outten C.E. Iron-sulfur cluster signaling: the common thread in fungal iron regulation. Curr. Opin. Chem. Biol. 2020; 55:189–201. 32234663 10.1016/j.cbpa.2020.02.008 PMC7237280

[84] Chen C. , Pande K., French S.D., Tuch B.B., Noble S.M. An iron homeostasis regulatory circuit with reciprocal roles in *Candida albicans* commensalism and pathogenesis. Cell Host Microbe. 2011; 10:118–135. 21843869 10.1016/j.chom.2011.07.005 PMC3165008

[85] Jung W.H. , Sham A., White R., Kronstad J.W. Iron regulation of the major virulence factors in the AIDS-associated pathogen *cryptococcus neoformans*. PLoS Biol. 2006; 4:e410. 17121456 10.1371/journal.pbio.0040410 PMC1637126

[86] Jung W.H. , Sánchez-León E., Kronstad J.W. Coordinated regulation of iron metabolism in *Cryptococcus neoformans* by GATA and CCAAT transcription factors: connections with virulence. Curr. Genet. 2021; 67:583–593. 33760942 10.1007/s00294-021-01172-5 PMC8428816

[87] Fuchs G. , Oren M. Writing and reading H2B monoubiquitylation. Biochim. Biophys. Acta. 2014; 1839:694–701. 24412854 10.1016/j.bbagrm.2014.01.002

[88] Lee K.K. , Florens L., Swanson S.K., Washburn M.P., Workman J.L. The deubiquitylation activity of Ubp8 is dependent upon Sgf11 and its association with the SAGA complex. Mol. Cell. Biol. 2005; 25:1173–1182. 15657442 10.1128/MCB.25.3.1173-1182.2005 PMC544014

[89] Lee K.K. , Swanson S.K., Florens L., Washburn M.P., Workman J.L. Yeast Sgf73/Ataxin-7 serves to anchor the deubiquitination module into both SAGA and Slik(SALSA) HAT complexes. Epigenetics Chromatin. 2009; 2:2. 19226466 10.1186/1756-8935-2-2 PMC2657900

[90] Köhler A. , Schneider M., Cabal G.G., Nehrbass U., Hurt E. Yeast Ataxin-7 links histone deubiquitination with gene gating and mRNA export. Nat. Cell Biol. 2008; 10:707–715. 18488019 10.1038/ncb1733

[91] Yang J. , Chen D., Matar K.A.O., Zheng T., Zhao Q., Xie Y., Gao X., Li M., Wang B., Lu G.D. The deubiquitinating enzyme MoUbp8 is required for infection-related development, pathogenicity, and carbon catabolite repression in *magnaporthe oryzae*. Appl. Microbiol. Biotechnol. 2020; 104:5081–5094. 32274561 10.1007/s00253-020-10572-5

[92] Steidl S. , Papagiannopoulos P., Litzka O., Andrianopoulos A., Davis M.A., Brakhage A.A., Hynes M.J. AnCF, the CCAAT binding complex of *Aspergillus nidulans*, contains products of the hapB, hapC, and hapE genes and is required for activation by the pathway-specific regulatory gene amdR. Mol. Cell. Biol. 1999; 19:99–106. 9858535 10.1128/mcb.19.1.99 PMC83869

[93] McNabb D.S. , Pinto I. Assembly of the Hap2p/Hap3p/Hap4p/Hap5p-DNA complex in *saccharomyces cerevisiae*. Euk. Cell. 2005; 4:1829–1839. 10.1128/EC.4.11.1829-1839.2005 PMC1287863 16278450

[94] Mendoza-Mendoza A. , Eskova A., Weise C., Czajkowski R., Kahmann R. Hap2 regulates the pheromone response transcription factor prf1 in *Ustilago maydis*. Mol. Microbiol. 2009; 72:683–698. 19400774 10.1111/j.1365-2958.2009.06676.x

[95] Singh R.P. , Prasad H.K., Sinha I., Agarwal N., Natarajan K. Cap2-HAP complex is a critical transcriptional regulator that has dual but contrasting roles in regulation of iron homeostasis in *Candida albicans*. J. Biol. Chem. 2011; 286:25154–25170. 21592964 10.1074/jbc.M111.233569 PMC3137088

[96] Ridenour J.B. , Bluhm B.H. The HAP complex in *Fusarium verticillio*ides is a key regulator of growth, morphogenesis, secondary metabolism, and pathogenesis. Fung. Genet. Biol. 2014; 69:52–64. 10.1016/j.fgb.2014.05.003 24875423

[97] Huber E.M. , Hortschansky P., Scheven M.T., Misslinger M., Haas H., Brakhage A.A., Groll M. Structural insights into cooperative DNA recognition by the CCAAT-binding complex and its bZIP transcription factor HapX. Structure. 2022; 30:934–946. 35472306 10.1016/j.str.2022.04.001

[98] Pelletier B. , Mercier A., Dur and M., Peter C., Jbel M., Beaudoin J., Labbé S. Expression of *Candida albicans* Sfu1 in fission yeast complements the loss of the iron-regulatory transcription factor Fep1 and requires Tup co-repressors. Yeast. 2007; 24:883–900. 17724773 10.1002/yea.1539

[99] Zhang N. , MohdZainudin N.A., Scher K., Condon B.J., Horwitz B.A., Turgeon B.G. Iron, oxidative stress, and virulence: roles of iron-sensitive transcription factor Sre1 and the redox sensor ChAp1 in the maize pathogen *Cochliobolus heterostrophus*. Mol. Plant Microbe. Interact. 2013; 26:1473–1485. 23980626 10.1094/MPMI-02-13-0055-R

[100] Bargaje R. , Alam M.P., Patowary A., Sarkar M., Ali T., Gupta S., Garg M., Singh M., Purkanti R., Scaria V. et al. Proximity of H2A.Z containing nucleosome to the transcription start site influences gene expression levels in the mammalian liver and brain. Nucleic Acids Res. 2012; 40:8965–8978. 22821566 10.1093/nar/gks665 PMC3467062

[101] Courtney A.J. , Kamei M., Ferraro A.R., Gai K., He Q., Honda S., Lewis Z.A. Normal patterns of histone H3K27 methylation require the histone variant H2A.Z in *Neurospora crassa*. Genetics. 2020; 216:51–66. 32651262 10.1534/genetics.120.303442 PMC7463285

[102] Berriri S. , Gangappa S.N., Kumar S.V. SWR1 Chromatin-remodeling complex subunits and H2A.Z have non-overlapping functions in immunity and gene regulation in *arabidopsis*. Mol. Plant. 2016; 9:1051–1065. 27131447 10.1016/j.molp.2016.04.003 PMC4938710

[103] Raisner R.M. , Hartley P.D., Meneghini M.D., Bao M.Z., Liu C.L., Schreiber S.L., Rando O.J., Madhani H.D. Histone variant H2A.Z marks the 5' ends of both active and inactive genes in euchromatin. Cell. 2005; 123:233–248. 16239142 10.1016/j.cell.2005.10.002 PMC2039754

[104] Keogh M.C. , Kim J.A., Downey M., Fillingham J., Chowdhury D., Harrison J.C., Onishi M., Datta N., Galicia S., Emili A. et al. A phosphatase complex that dephosphorylates gammaH2AX regulates DNA damage checkpoint recovery. Nature. 2006; 439:497–501. 16299494 10.1038/nature04384

[105] Alatwi H.E. , Downs J.A. Removal of H2A.Z by INO80 promotes homologous recombination. EMBO Rep. 2015; 16:986–994. 26142279 10.15252/embr.201540330 PMC4552491

[106] Gursoy-Yuzugullu O. , Ayrapetov M.K., Price B.D. Histone chaperone Anp32e removes H2A.Z from DNA double-strand breaks and promotes nucleosome reorganization and DNA repair. Proc. Natl. Acad. Sci. U.S.A. 2015; 112:7507–7512. 26034280 10.1073/pnas.1504868112 PMC4475971

[107] Talbert P.B. , Henikoff S. Environmental responses mediated by histone variants. Trends Cell Biol. 2014; 24:642–650. 25150594 10.1016/j.tcb.2014.07.006

[108] Cortijo S. , Charoensawan V., Brestovitsky A., Buning R., Ravarani C., Rhodes D., van Noort J., Jaeger K.E., Wigge P.A. Transcriptional regulation of the ambient temperature response by H2A.Z nucleosomes and HSF1 transcription factors in *arabidopsis*. Mol. Plant. 2017; 10:1258–1273. 28893714 10.1016/j.molp.2017.08.014 PMC6175055

[109] Dong Q. , Wang Y., Qi S., Gai K., He Q., Wang Y. Histone variant H2A.Z antagonizes the positive effect of the transcriptional activator CPC1 to regulate catalase-3 expression under normal and oxidative stress conditions. Free Radic. Biol. Med. 2018; 121:136–148. 29738831 10.1016/j.freeradbiomed.2018.05.003

[110] Creyghton M.P. , Markoulaki S., Levine S.S., Hanna J., Lodato M.A., Sha K., Young R.A., Jaenisch R., Boyer L.A. H2AZ is enriched at polycomb complex target genes in ES cells and is necessary for lineage commitment. Cell. 2008; 135:649–661. 18992931 10.1016/j.cell.2008.09.056 PMC2853257

[111] Coleman-Derr D. , Zilberman D Deposition of histone variant H2A.Z within gene bodies regulates responsive genes. PLoS Genet. 2012; 8:e1002988. 23071449 10.1371/journal.pgen.1002988 PMC3469445

[112] Zhang K. , Xu W., Wang C., Yi X., Zhang W., Su Z. Differential deposition of H2A.Z in combination with histone modifications within related genes in *Oryza sativa* callus and seedling. Plant J. 2017; 89:264–277. 27643852 10.1111/tpj.13381

[113] Whittle C.M. , McClinic K.N., Ercan S., Zhang X., Green R.D., Kelly W.G., Lieb J.D. The genomic distribution and function of histone variant HTZ-1 during *C. elegans* embryogenesis. PLoS Genet. 2008; 4:e1000187. 18787694 10.1371/journal.pgen.1000187 PMC2522285

[114] Kumar S.V. , Wigge P.A. H2A.Z-containing nucleosomes mediate the thermosensory response in *Arabidopsis*. Cell. 2010; 140:136–147. 20079334 10.1016/j.cell.2009.11.006

[115] Attarian R. , Hu G., Sánchez-León E., Caza M., Croll D., Do E., Bach H., Missall T., Lodge J., Jung W.H. et al. The Monothiol glutaredoxin Grx4 regulates iron homeostasis and virulence in *Cryptococcus neoformans*. mBio. 2018; 9:e02377-18. 30514787 10.1128/mBio.02377-18 PMC6282196

[116] Martínez-Pastor M.T. , Puig S. Adaptation to iron deficiency in human pathogenic fungi. Biochim. Biophys. Acta. Mol. Cell Res. 2020; 1867:118797. 32663505 10.1016/j.bbamcr.2020.118797

[117] Misslinger M. , Scheven M.T., Hortschansky P., López-Berges M.S., Heiss K., Beckmann N., Heigl T., Hermann M., Krüger T., Kniemeyer O. et al. The monothiol glutaredoxin GrxD is essential for sensing iron starvation in *Aspergillus fumigatus*. PLoS Genet. 2019; 15:e1008379. 31525190 10.1371/journal.pgen.1008379 PMC6762210

[118] Alkafeef S.S. , Lane S., Yu C., Zhou T., Solis N.V., Filler S.G., Huang L., Liu H. Proteomic profiling of the monothiol glutaredoxin Grx3 reveals its global role in the regulation of iron dependent processes. PLoS Genet. 2020; 16:e1008881. 32525871 10.1371/journal.pgen.1008881 PMC7319344

