# Supplementary material for: HapX-mediated H2B deub1 and SreA-mediated H2A.Z deposition coordinate in fungal iron resistance
Source: Nucleic Acids Res. 2023 Aug 31;51(19):10238–60. doi: 10.1093/nar/gkad708 (PMC10602907; doi:10.1093/nar/gkad708)
Supplement: gkad708_Supplemental_Files [file gkad708_supplemental_files.zip › Supplementary Figures 20230724.pdf]

SUPPORTING INFORMATION:

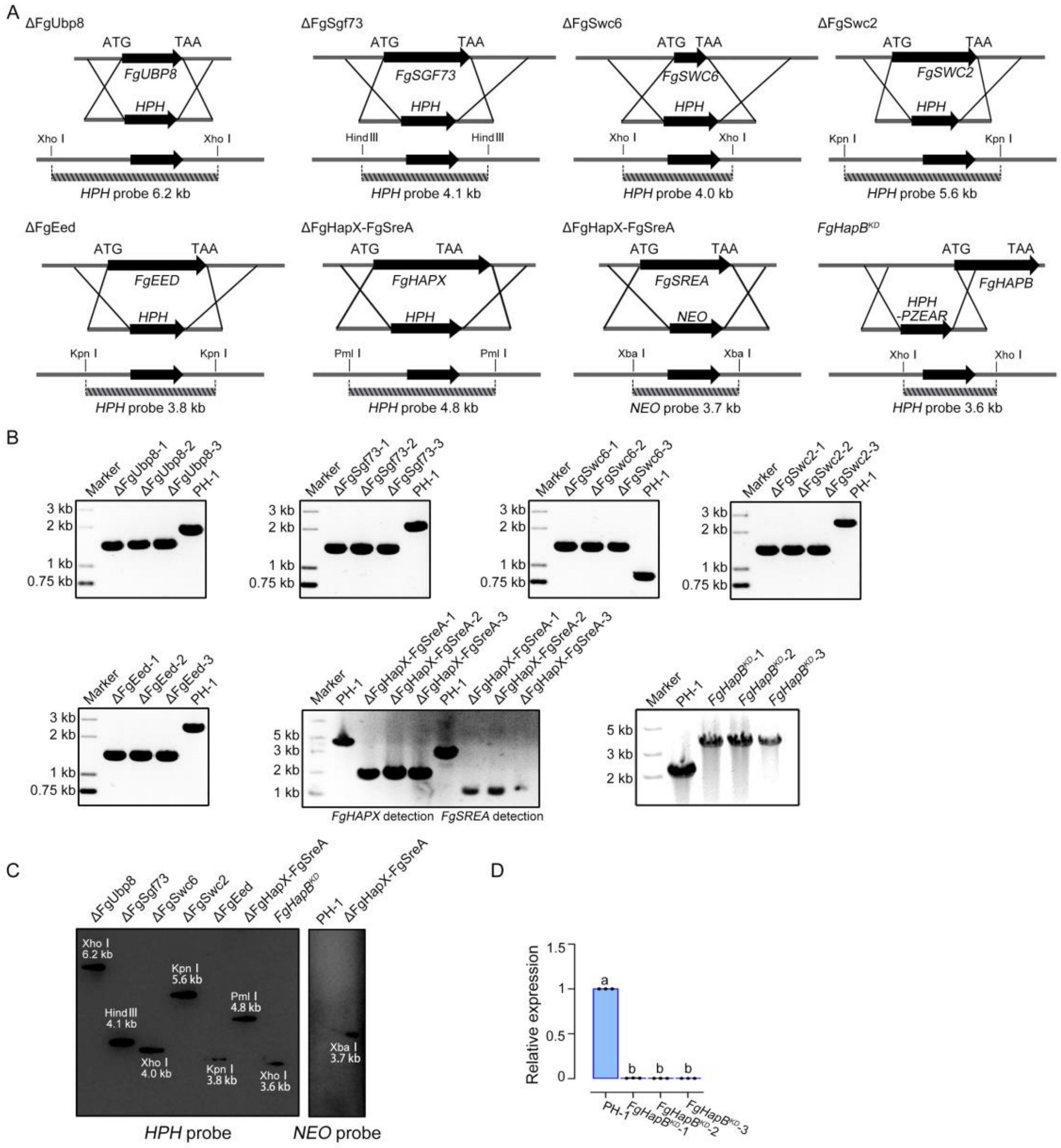

Supplementary Figure S1. Construction and identification of deletion and knock-down mutants.

(**A**) Gene replacement strategy and Southern blot information for the deletion mutants ΔFgUbp8, ΔFgSgf73, ΔFgSwc6, ΔFgSwc2, ΔFgEed, double deletion mutant ΔFgHapX-FgSreA, and knock-down mutant *FgHapB*^{KD}. Restriction sites and probes information were indicated for each gene. (**B**) PCR identification for the above mutants. (**C**) Southern blot analyses of the above mutants. (**D**) The expression level of

FgHAPB in the wild type and $FgHap^{KD}$ was determined by qRT-PCR assays. The *FgACTIN* gene was used as the internal control for normalization.

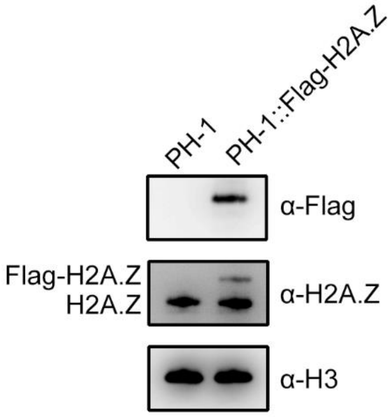

Supplementary Figure S2. Specificity validation of H2A.Z polyclonal antibody

Western blot assays of H2A.Z or Flag in PH-1 and PH-1::Flag-H2A.Z stains. H2A.Z polyclonal antibody specifically recognizes H2A.Z in PH-1, and recognizes Flag-H2A.Z and H2A.Z in PH-1::Flag-H2A.Z. Flag antibody can only recognize Flag-H2A.Z in PH-1::Flag-H2A.Z. H3 level detected with anti-H3 antibody was conducted as the protein loading reference.

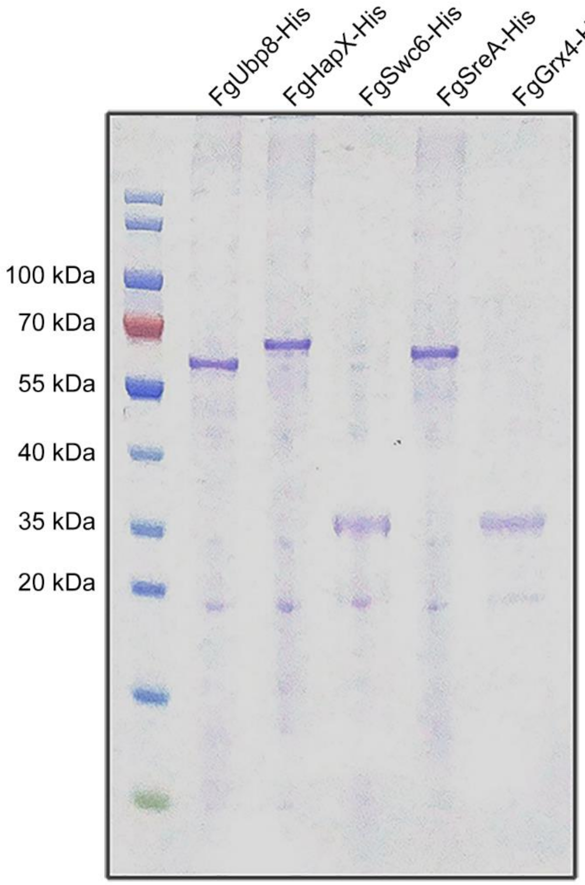

Supplementary Figure S3. Expression and purification of His-tagged FgUbp8, FgHapX, FgSwc6, FgSreA and FgGrx4 proteins.

Fusion proteins containing a 6×His tag were expressed in *E. coli* BL21 upon induction for 6 hours at 30 ℃ in the presence of 0.5 mM isopropyl-β-d-thiogalactopyranoside, and then purified by using FPLC system purification. Coomassie Brilliant Blue G-250 staining showed the protein samples resolved by SDS-PAGE.

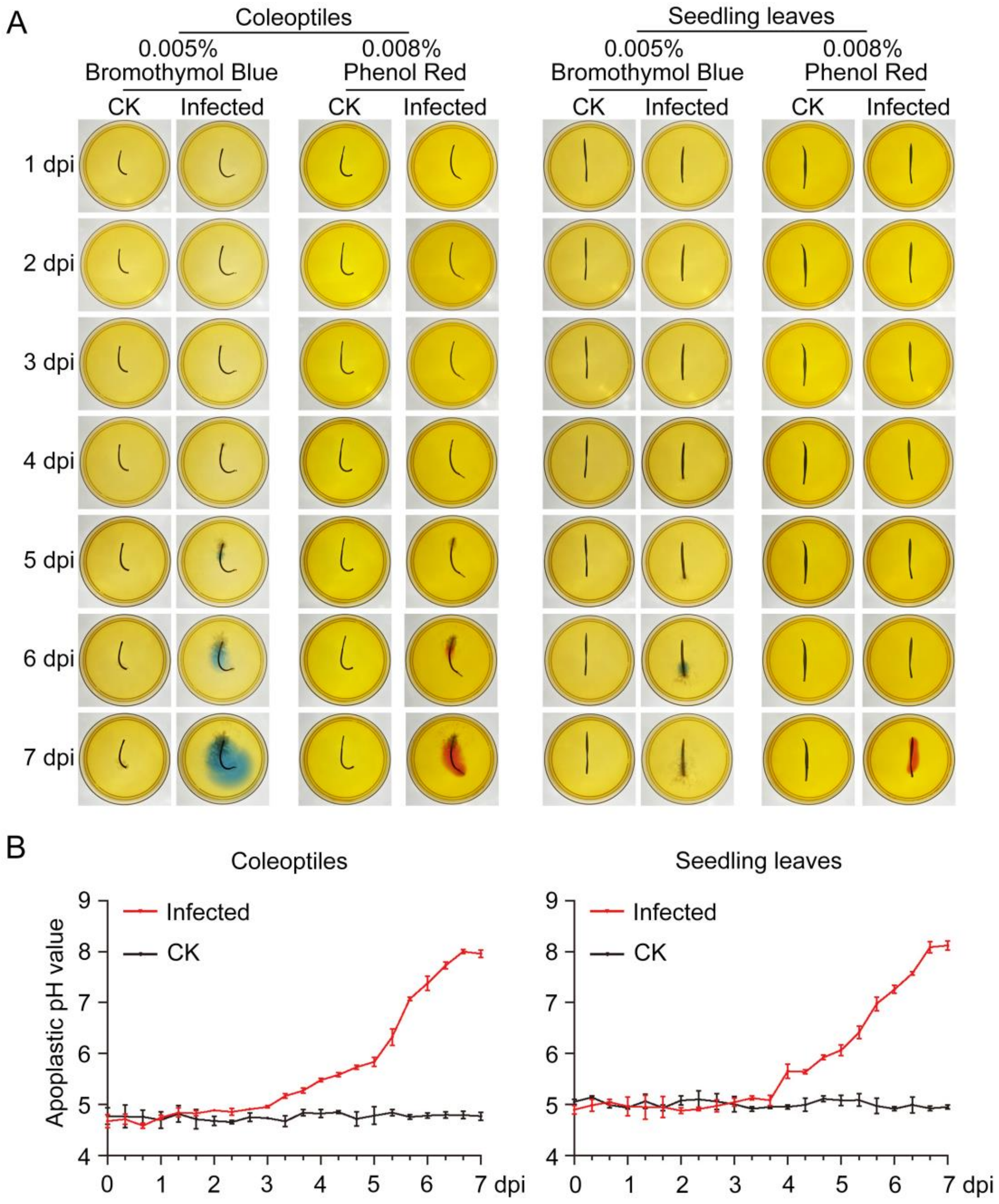

Supplementary Figure S4. *F. graminearum* induces extracellular alkalinization in wheat coleoptiles and seedling leaves.

(**A**) Wheat coleoptiles and seedling leaves were placed on water agar plates (adjusted to pH 5.0 containing 0.005% [w/v] bromothymol blue or 0.008% [w/v] phenol red), and then inoculated with the conidial suspension of *F. graminearum* wild-type strain PH-1 or water as a control. Plates were imaged each 7 days. Bromothymol blue staining: bright yellow indicates pH < 5.2, deep purple indicates pH > 6.8. Phenol red staining: bright yellow indicates pH < 6.8, red indicates pH>8.0.

The experiment was repeated independently three times. (B) The pH levels in apoplastic fluids of wheat coleoptiles and seedling leaves at different time points after inoculation with *F. graminearum*. Mean and standard deviation were estimated with data from three independent biological replicates.

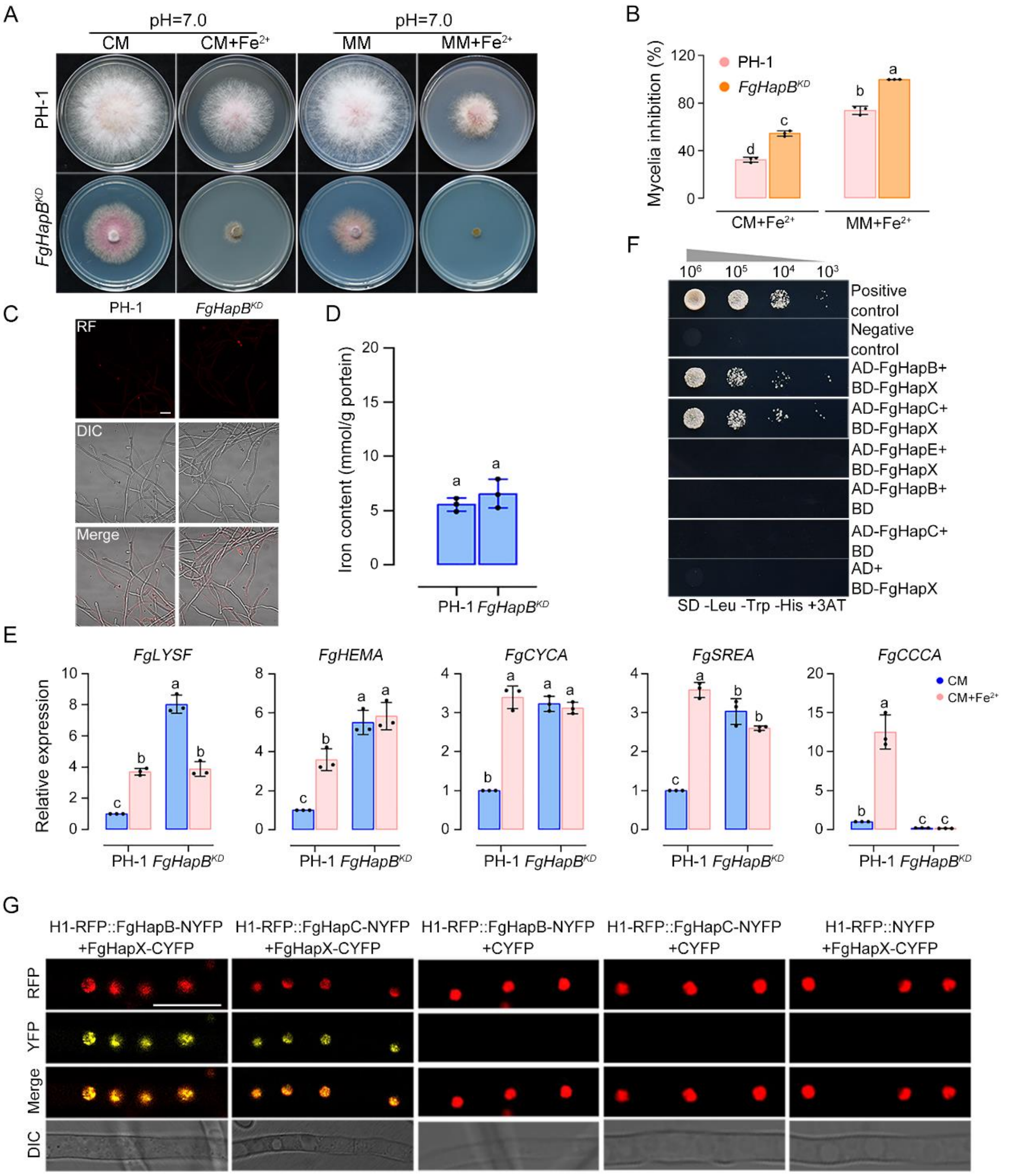

Supplementary Figure S5. The heterotrimer CCAAT-binding complex (CBC) is required for the function of FgHapX in iron homeostasis regulation.

(**A**, **B**) Knock-down mutant *HapB*^{KD} caused increased sensitivity to iron excess. Colony morphology was observed (**A**), and mycelial inhibition was determined (**B**) after growth on CM with or without 10 mM Fe_2SO_4 for 3 d. (**C**, **D**) The *HapB*^{KD} displayed unaltered iron content compared to the wild type. Iron content of each strain was determined by a laser scanning microscope with 5 μM fluorescent iron-binding dye FeRhoNox-1 (**C**) or colorimetric ferrozine-based assay (**D**) after culture in CM

for 36 hr. Bar = 20 μm. Fluorescence intensity was further evaluated by line-scan graph analysis, and the horizontal axis indicates the distance. (**E**) Knocking down of FgHapB altered the transcription of iron utilization and storage genes, and *FgSREA*. The expression level of each iron homeostasis gene in the wild type (PH-1) in CM without iron excess treatment was set to 1 and the *FgACTIN* gene was used as the internal control for normalization. (**F**) FgHapX interacts with FgHapB and FgHapC in Y2H assays. Serial dilutions of yeast cells (cells/ml) transferred with the bait and prey constructs were assayed for growth on SD-Leu-Trp-His+3AT plates. A pair of plasmids pGBKT7 (BD)-53 and pGADT7 (AD)-T was used as a positive control. A pair of plasmids pGBKT7-Lam and pGADT7-T was used as a negative control. (**G**) FgHapX interacts with FgHapB and FgHapC in BiFC assays. Pairs of constructs FgHapB-NYFP+CYFP, FgHapC-NYFP+CYFP, FgHapX-CYFP+NYFP were used as negative controls. YFP signals were observed using confocal microscopy. Bar = 10 μm. In (**B**, **D**, **E**), mean and standard deviation were estimated with data from three independent biological replicates (marked with black or red dots, n = 3). Different letters indicate statistically significant differences according to the one-way ANOVA test ($p < 0.05$).

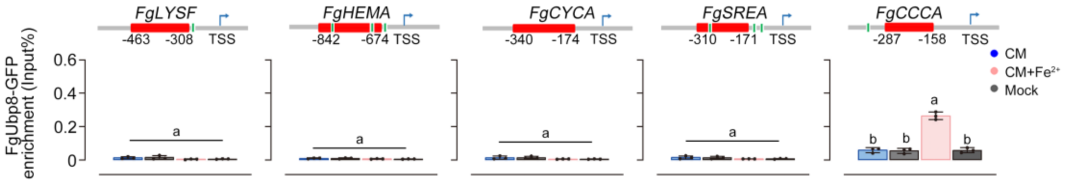

Supplementary Figure S6. ChIP-qPCR analyses revealed that FgUbp8 was not enriched at the promoters of iron utilization genes and *FgSREA*.

ChIP-qPCR assays revealed that FgUbp8-GFP was not enriched at *FgLYSF*, *FgHEMA*, *FgCYCA* and *FgSREA* promoter with or without iron excess treatment. The gene *FgCCCA* used as a positive control. The input-DNA and ChIP-DNA samples were quantified by quantitative PCR assays with corresponding primer pairs (Supplementary Table S1). ChIP signals are shown as the percentages of input and mock-DNA incubated with anti-IgG antibody as a control. Mean and standard deviation were estimated with data from three independent biological replicates (marked with black or red dots, n = 3). Different letters indicate statistically significant differences according to the one-way ANOVA test ($p < 0.05$).

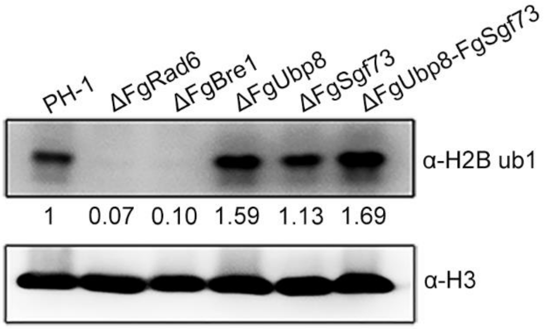

Supplementary Figure S7. FgSgf73 is not involved in the regulation of H2B deub1.

Western blot assays of global H2B ub1 level in the wide type, ΔFgUbp8, ΔFgSgf73 and ΔFgUbp8-FgSgf73. H2B ub1 level was detected with the anti-H2B ub1 antibody. H3 level detected with anti-H3 antibody was conducted as the protein loading reference. The intensities of the western blotting bands were quantified with the program ImageJ. Values on the bars are the intensity of detected protein band relative to that of H3 band.

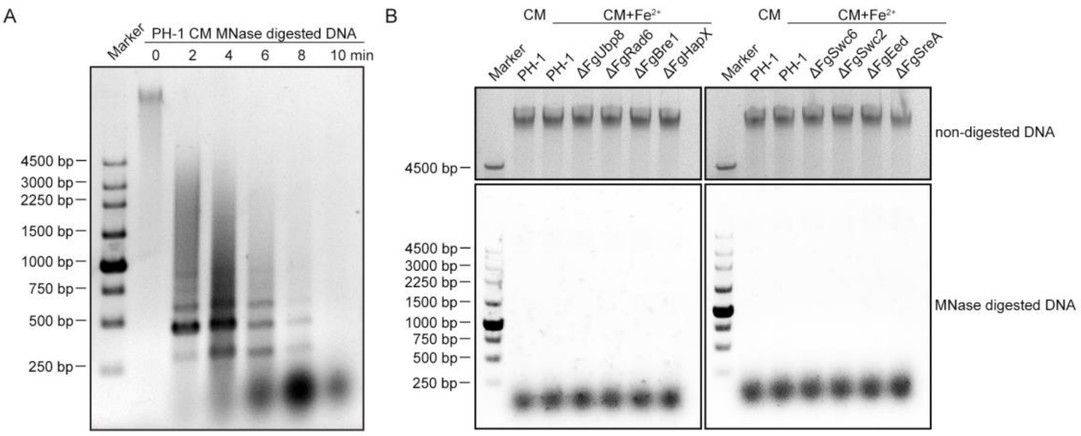

Supplementary Figure S8. Different DNAs achieved similar MNase digestion level.

(**A**) Agarose gels demonstrate MNase digestion ladders. To obtain mono-nucleosomes from partial digestion, DNA fragments (size between 100 bp and 200 bp) from the lane of “8 min” achieved high digestion efficiency. (**B**) The gels showed similar MNase digestion levels of above different DNA samples after 8 min digestion.

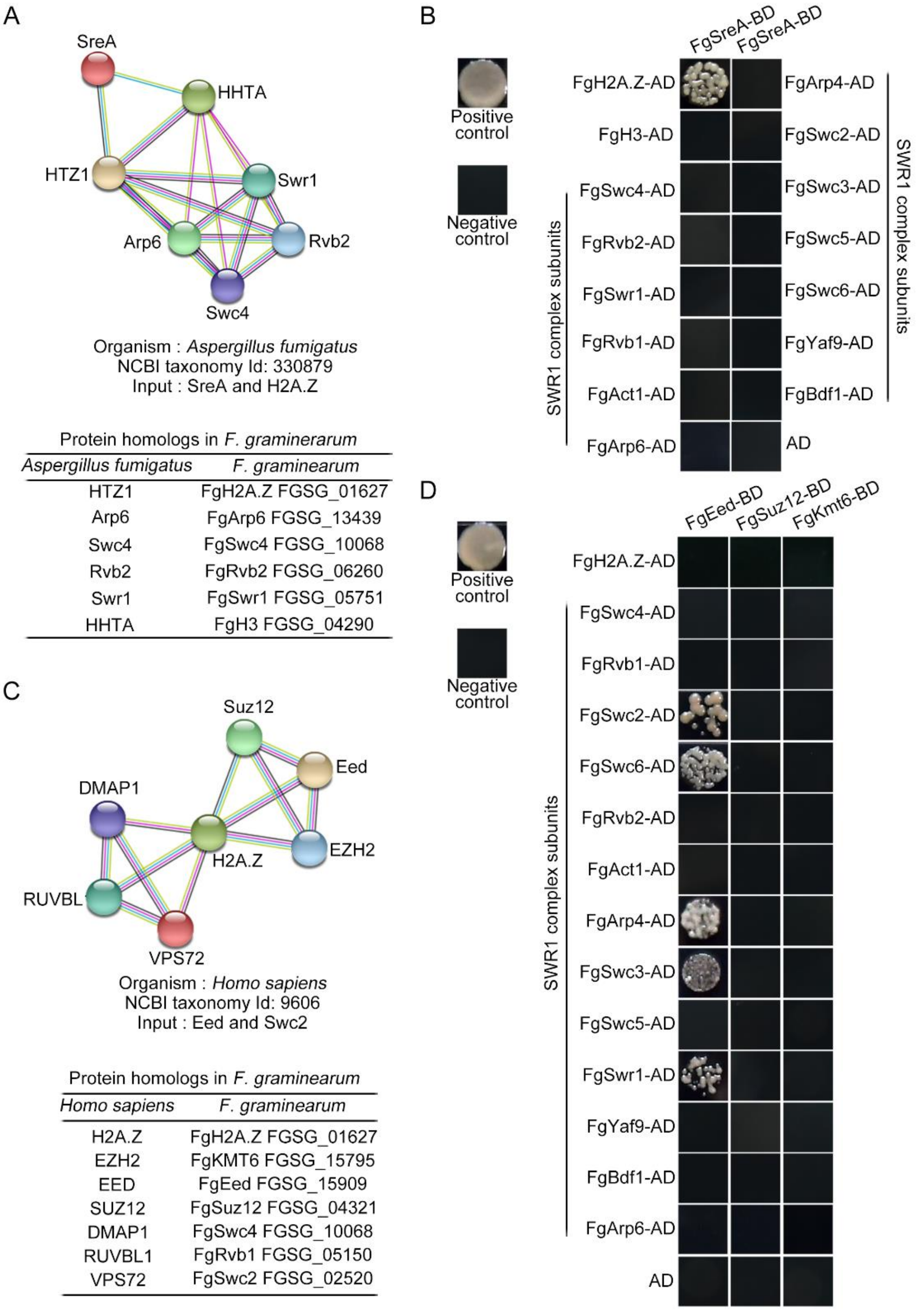

Protein homologs in *F. graminerarum*

Aspergillus fumigatus	*F. graminearum*
HTZ1	FgH2A.Z FGSG_01627
Arp6	FgArp6 FGSG_13439
Swc4	FgSwc4 FGSG_10068
Rvb2	FgRvb2 FGSG_06260
Swr1	FgSwr1 FGSG_05751
HHTA	FgH3 FGSG_04290

Protein homologs in *F. graminearum*

Homo sapiens	*F. graminearum*
H2A.Z	FgH2A.Z FGSG_01627
EZH2	FgKMT6 FGSG_15795
EED	FgEed FGSG_15909
SUZ12	FgSuz12 FGSG_04321
DMAP1	FgSwc4 FGSG_10068
RUVBL1	FgRvb1 FGSG_05150
VPS72	FgSwc2 FGSG_02520

Supplementary Figure S9. The prediction and confirmation of potential interacing protein pairs.

(**A**) A list of potential FgSreA-interacting proteins predicted using STRING database 11.5 (https://string-db.org). (**B**) FgSreA interacts only with FgH2A.Z in Y2H assays. Serial dilutions of yeast cells (cells/ml) transferred with the bait and prey constructs were assayed for growth on SD-Leu-Trp-His+3AT plates. The plasmid pair pGBKT7

(BD)-53 and pGADT7 (AD)-T used as a positive control. The plasmid pair pGBKT7-Lam and pGADT7-T used as a negative control. (**C**) A list of potential FgH2A.Z-interacting proteins predicted using STRING database 11.5 (https://string-db.org). (**D**) The SWR1 complex interacts with the PRC2 complex in *F. graminearum* in Y2H assays. Serial dilutions of yeast cells (cells/ml) transferred with the bait and prey constructs were assayed for growth on SD-Leu-Trp-His+3AT plates. The plasmid pair pGBKT7-53 and pGADT7-T used as a positive control. The plasmid pair pGBKT7-Lam and pGADT7-T used as a negative control.

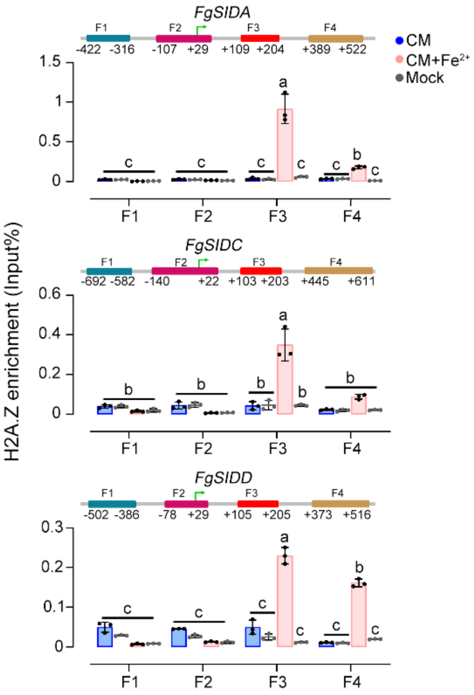

Supplementary Figure S10. ChIP-qPCR assays show the enrichment of H2A.Z around the iron acquisition genes TSS regions upon iron excess.

The enrichment of H2A.Z around the TSS (transcription start site) regions of iron acquisition genes in ChIP-qPCR assays. The input-DNA and ChIP-DNA samples were quantified by quantitative PCR assays with a set of primer pairs. The enrichment localization for each gene was shown. ChIP signals are shown as the percentages of input and mock-DNA incubated with anti-IgG antibody as a control. Mean and standard deviation were estimated with data from three independent biological replicates (marked with dots in different color, n = 3). Different letters indicate statistically significant differences according to the one-way ANOVA test ($p < 0.05$).

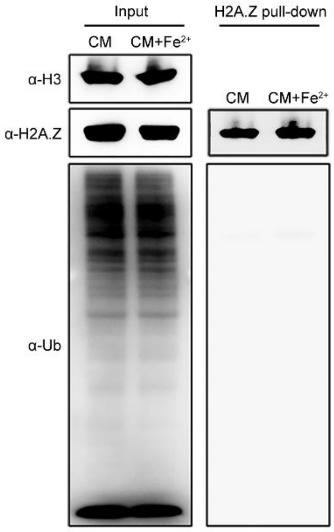

Supplementary Figure S11. FgH2A.Z is not subject to ubiquitination under iron excess treatment.

The protein samples detected with anti-H3 antibody were used as a reference.

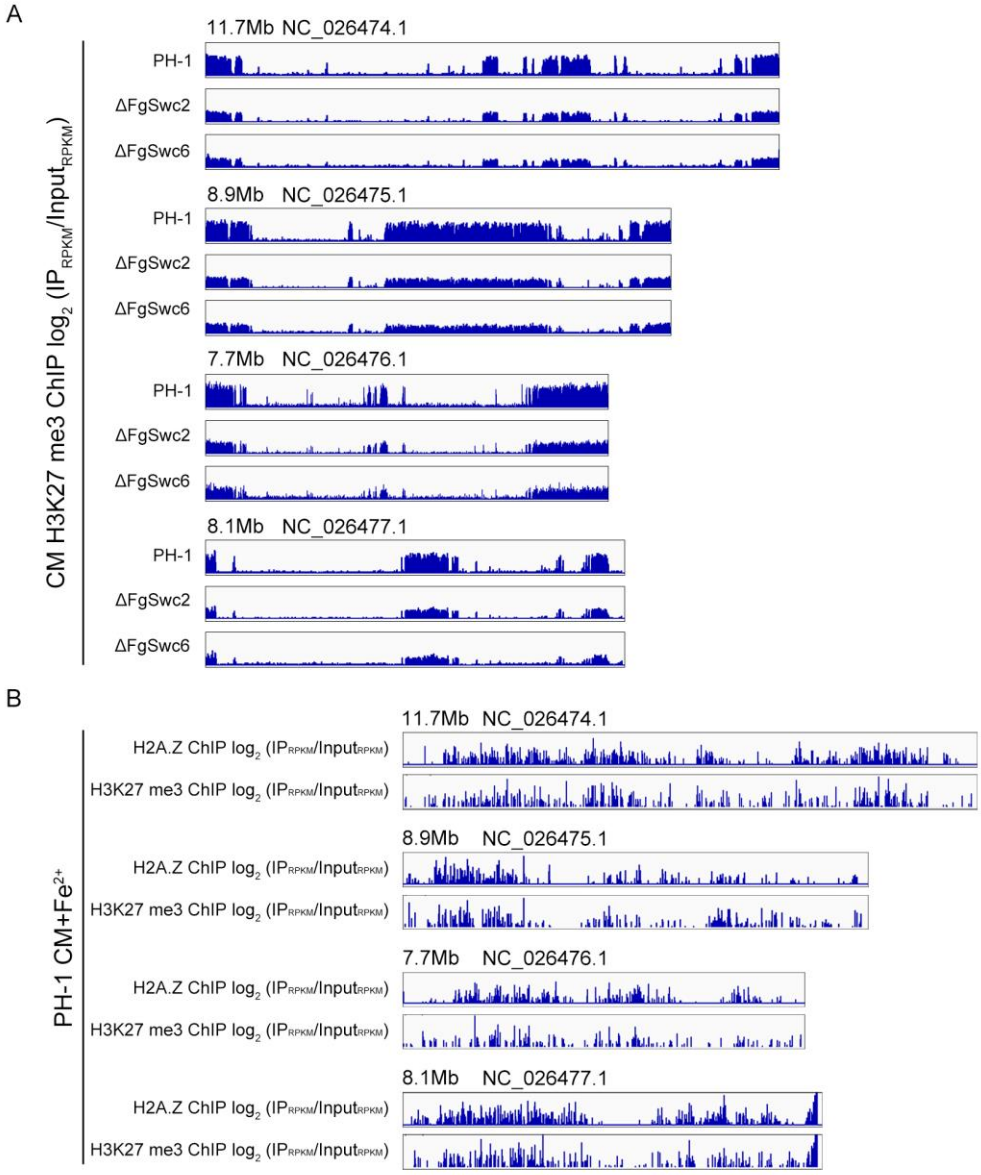

Supplementary Figure S12. Genome-wide distribution of H3K27 me3 and H2A.Z.

(**A**) Genome browser view of H3K27 me3 peaks in PH-1, ΔFgSwc2 and ΔFgSwc6 in CM culture. (**B**) Genome browser view of H2A.Z and H3K27 me3 peaks in PH-1 under iron excess treatment.

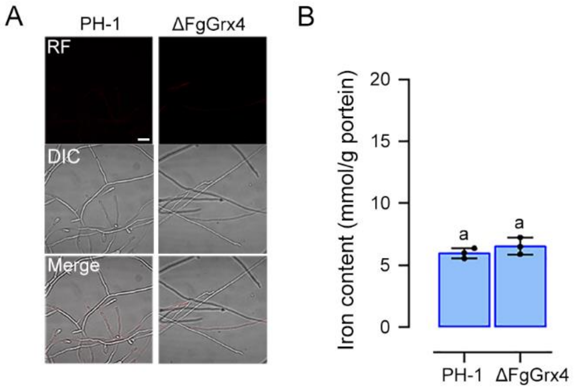

Supplementary Figure S13. Deletion of FgGrx4 did not change intracellular iron content.

(**A**, **B**) The ΔFgGrx4 displayed unaltered iron content compared to the wild type. Iron content of each strain was determined by a laser scanning microscope with 5 μM fluorescent iron-binding dye FeRhoNox-1 (**A**) or colorimetric ferrozine-based assay (**B**) after culture in CM for 36 hr. Bar = 20 μm. Fluorescence intensity was further evaluated by line-scan graph analysis, and the horizontal axis indicates the distance. Mean and standard deviation were estimated with data from three independent biological replicates (marked with dots in different color, n = 3). Different letters indicate statistically significant differences according to the one-way ANOVA test ($p < 0.05$).

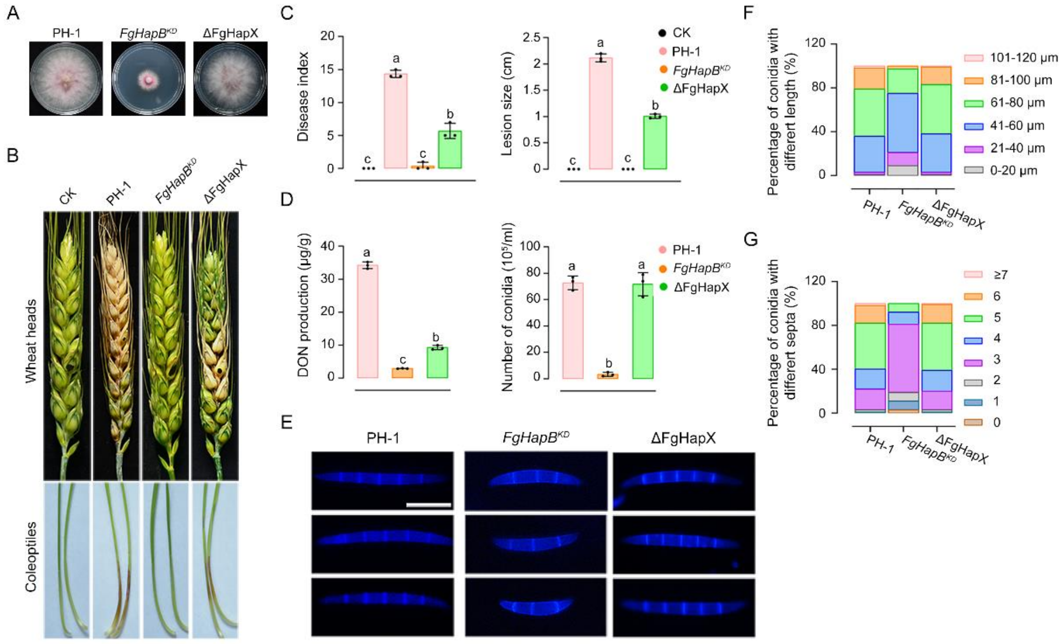

Supplementary Figure S14. Comparison of phenotypic defects between *FgHapB*^{KD} and ΔFgHapX.

(**A**) *FgHapB*^{KD} showed reduced mycelial growth on CM medium for 3 d. (**B**, **C**) Knocking down of FgHapB and deletion of FgHapX both caused significantly reduced virulence on wheat heads and coleoptiles. Representative images of wheat heads were photographed (**B**), and disease index was calculated (**C**) at 15 dpi. The inoculated site on each wheat head was labeled with a black dot. Representative images of coleoptiles were photographed (**B**), and lesion sizes were measured (**C**) at 4 dpi. (**D**) The *FgHapB*^{KD} exhibited more serious defects in deoxynivalenol (DON) production and asexual development than ΔFgHapX. Conidia were quantified using a hemacytometer after incubation of each strain in CMC for 4 days. (**E**) Differential interference contrast (DIC) images of conidia stained with calcofluor white (CFW) were captured with an electron microscopy. Bar = 20 μm. (**F**, **G**) Comparisons in conidial length (**F**) and septum number (**G**) among the above strains. A total of 100 conidia were examined for each strain. Mean and standard deviation were estimated with data from three independent biological replicates (marked with black or red dots, n = 3). Different letters indicate statistically significant differences according to the one-way ANOVA test ($p < 0.05$).

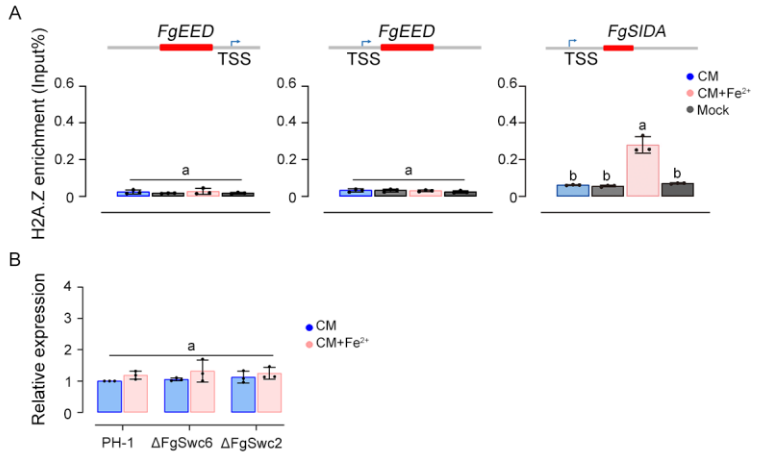

Supplementary Figure S15. FgH2A.Z was not enriched at *FgEED* and did not affect transcription.

(**A**) ChIP-qPCR assays revealed that FgH2A.Z was not enriched at the promoter and +1 nucleosome region of *FgEED* with or without iron excess treatment. The gene *FgSIDA* used as a positive control. ChIP signals are shown as the percentages of input and mock-DNA incubated with anti-IgG antibody was used as a control. (**B**) Transcription of *FgEED* in PH-1, ΔFgSwc6 and ΔFgSwc2, with or without iron excess treatment in qRT-PCR assays. The expression level of each gene in PH-1 in CM without iron excess treatment was set to 1 and the *FgACTIN* gene was used as the internal control for normalization. Mean and standard deviation were estimated with data from three independent biological replicates (marked with black or gray dots, n = 3). Different letters indicate statistically significant differences according to the one-way ANOVA test ($p < 0.05$).
